# A systematic review of global health capacity building initiatives in low-to middle-income countries in the Middle East and North Africa region

**DOI:** 10.1186/s12992-020-00585-0

**Published:** 2020-07-03

**Authors:** Hady Naal, Maria El Koussa, Melissa El Hamouch, Layal Hneiny, Shadi Saleh

**Affiliations:** 1 Global Health Institute at the American University of Beirut, Faculty of Health Sciences at the American University of Beirut, Beirut, Lebanon; 2 Saab Medical Library at the American University of Beirut, Beirut, Lebanon

**Keywords:** Capacity building, Global Health, Middle East and North Africa, Low to middle income countries

## Abstract

**Introduction:**

Low-and Middle-Income Countries (LMICs) in the Middle East and North Africa (MENA) region are facing increasing global health challenges with a reduced ability to manage them. Global Health Capacity Building (GHCB) initiatives have the potential to improve health workforce performance and health outcomes, however little is known about the GHCB topics and approaches implemented in this region. This is the first systematic review of GHCB initiatives among LMICs in the MENA region.

**Methods:**

An academic database search of Medline (OVID), PubMed, Scopus, Embase.com, and Open Grey was conducted for articles published between January 2009 and September 2019 in English. Next, a grey literature search following a recommended search framework was conducted. Reviewed records addressed a global health topic, had a capacity building component, looked at specific learning outcomes, and reflected an LMIC in the MENA. Primary outcomes included country, topic, modality, pedagogy, and population.

**Results:**

Reports of GHCB initiatives were retrieved from grey sources (73.2%) and academic sources (26.8%). Most GHCB initiatives were mainly conducted face-to-face (94.4%) to professional personnel (57.5%) through a theoretical pedagogical approach (44.3%). Dominant global health themes were non-communicable diseases (29.2%), sexual and reproductive health (18.4%), and mental health (14.5%). When matched against the Global Burden of Disease data, important gaps were found regarding the topics of GHCB initiatives in relation to the region’s health needs. There were limited reports of GHCB initiatives addressing conflict and emergency topics, and those addressing non-communicable disease topics were primarily reported from Egypt and Iran.

**Conclusion:**

Innovative and practicum-based approaches are needed for GHCB initiatives among LMICs in the MENA region, with a focus on training community workers. Regional and country-specific analyses of GHCB initiatives relative to their health needs are discussed in the manuscript based on the results of this review.

## Introduction

Over the past few decades, the Middle East and North Africa (MENA) region has made progress in reducing rates of disease, injury, and premature death [1]. Although countries in the MENA region are prolonging the lives of their populations and limiting mortality rates, this region continues to experience significant disease burdens, coupled with a reduced capability to manage them [1, 2]. Low-to Middle-Income Countries (LMICs) in specific tend to face greater health challenges among countries in the region, largely due to their decreased resources in comparison to Higher-Income Countries (HIC) in the region. In recent years, this has been exacerbated by conflicts occurring in many countries that contributed not only in limited investment towards building the health workforce to meet the health and conflict-related needs, but additionally to an exodus of a large number of experienced health workers, further straining limited resources [3]. Although the MENA region has the third lowest density of doctors and nurses, it experiences one of the highest disease burdens after Southeast Asia and Sub-Saharan Africa [4].

Limited access to education, training, mentoring, and continuous professional development are leading contributing factors that undermine the performance and commitment of healthcare workers [5, 6]. Healthcare workers are personnel who engage in service provision or decision-making to improve health in given settings. As an example, many schools and institutions that provide health-related training and education in LMICs face important shortcomings in equipment, physical space, curricula, training materials, faculty, staff, and funding [7, 8]. These challenges suppress efforts to improve the quality of training and to expand the diversity and number of health-related programs, which negatively affect their responses to global health threats [7]. In many cases, this also makes it challenging for them to deliver even basic health services [5]. In order to improve health outcomes among LMICs in the MENA region, it is crucial to increase the number of the healthcare workforce and to strengthen their competencies through engaging approaches. Evidence suggests that an effectively trained and deployed health workforce is positively associated with addressing many health challenges, and has the potential to improve health outcomes [9]. Furthermore, ensuring equitable access to a skilled health workforce is a critical element to achieving the health or health-related Sustainable Development Goals (SDGs). This is especially true for LMICs that lack the necessary resources to mobilize efficiently and effectively trained and distributed human resources for health [10].

Global Health Capacity Building (GHCB) initiatives aim to enhance the capabilities of individuals, organizations, and communities to work in or manage global health-related topics [11]. The field of global health is multidisciplinary, and it encompasses health issues that transcend national boundaries [12]. For example, research, practice, and education in global health may cover topics such as communicable and infectious diseases, mental health and substance, traffic and conflict-related injuries, chronic non-communicable diseases, among others [12]. Implementing GHCB initiatives is a recommended, effective, and efficient strategy to enhance the capabilities of health workers in responding to related challenges [13]. GHCB initiatives enhance the skills, knowledge, and practices of professional and non-professional health workers, which may ultimately affect overall health outcomes in a given setting [13, 14]. Despite the importance and urgency of the aforementioned, the characteristics and focus areas of GHCB efforts conducted in the MENA region among LMICs have not been documented.

The aim of the present study is to provide the first systematic review of GHCB initiatives delivered in LMICs within the MENA region. Given that GHCB is essential to improving the competency and performance of the health workforce particularly within low-resource settings, this study is important to elucidate the GHCB topics and related approaches currently being addressed in relation to health challenges in the MENA region. This is an essential step to summarize the state of the field, and to identify related strengths and weaknesses.

## Methods

### Search strategy

Multiple search strategies were employed in this systematic review following the Preferred Reporting Items for Systematic Reviews and Meta-Analyses (PRISMA) in order to identify GHCB initiatives implemented among LMICs in the MENA region. This included an electronic academic database search, and a thorough grey literature mapping search. The latter was based on a WHO mapping framework which is a recommended approach developed by an authoritative source to conduct a mapping exercise. In both search strategies, articles had to reflect a GHCB initiative conducted in a LMIC in the MENA region. According to the World Bank, these countries include Algeria, Djibouti, Egypt, Iran, Iraq, Jordan, Lebanon, Libya, Morocco, Syria, Tunisia, West Bank and Gaza, and Yemen [15]. We used the World Bank classification for countries in the MENA region because it is a commonly used reference to locate countries in specific geographical regions [15]. Finally, we used the global burden of disease data for priority benchmarking throughout our analysis because it is the most widely used authoritative reference for disease rates globally and regionally.

#### Academic database

An electronic database search was conducted by a medical librarian (LH) using the following academic databases: Medline (OVID), PubMed, Scopus, and Embase.com, and Open Grey. The three concepts were “Global Health”, “Capacity Building”, and “Middle East and North Africa”, and included terms such as “courses”, “webinars”, “training”, “education”, “public health” among others. The full search strategy is reported in Additional file 1. Articles were included if they were qualitative, quantitative, and mixed-methods studies written in English and published between January 2009 and September 2019. Articles had to reflect GHCB initiatives conducted in a LMIC in the MENA region (see Table 1 for definitions). Although the field of global health encompasses leadership, management, and communication programs among others, in this review we only captured those that were explicitly health-related. Articles were excluded if they did not meet these criteria, or if they did not cover a global health topic, did not provide examples or cases about capacity building approaches, and were not conducted in a LMIC in the MENA region. Editorials, opinion pieces, letters to the editor, conference abstracts, study protocols, and press releases were excluded.

**Table 1:** Definition of Key Terms

Key Terms	Definitions
Global Health	“Health problems, issues, and concerns that transcend national boundaries, which may be influenced by circumstances or experiences in other countries, and which are best addressed by cooperative actions and solutions”. [16]
Capacity Building	The development of knowledge, skills, commitment, structures, systems, and leadership to enable effective health promotion … [with] actions to improve health at three levels: the advancement of knowledge and skills among practitioners; the expansion of support and infrastructure for health promotion in organizations, and; the development of cohesiveness and partnerships for health in communities. [17]
Population Groups	Professional Personnel have formal education and/or training in health fields such as doctors, researchers, nurses and so on. Community Workers have not received formal education and/or training but have one or more qualifications in related health fields to practice within their community. Examples include community health workers, community nurses and so on. General Public (e.g. community members, parents of school students etc. …) includes individuals who do not have formal education and/or training, and who do not practice in any area related to global health
Pedagogic Approach	Theory: Initiatives classified as training, workshop, course, or fellowship without further details on the approach. Interactive: Initiatives that included 1 or more combinations of presentations, group work, activities, participatory approaches, interactive discussions, open discussions, practical examples, role play, simulations etc.,,,. Practical: Initiatives that included a practical or technical training.
LMICs in MENA	Countries include Algeria, Djibouti, Egypt, Iran, Iraq, Jordan, Lebanon, Libya, Morocco, Syria, Tunisia, West Bank and Gaza Strip, and Yemen.
Global Health Topics Reviewed	Communicable Diseases (Anti-microbial resistance, immunization, malaria, other communicable diseases). Non-Communicable Diseases: (Cancer, diabetes, diarrhoea, heart failure, hypertension, and nutrition). Mental Health (General mental health topics, substance use, and psychosocial support) Sexual and Reproductive Health (Sexually transmitted infections, maternal and reproductive health, gender-based violence, and sexual harassment). Health System: (Health safety, workforce development, health services, and health research). Child health (Not specified). Disaster & Emergency Preparedness (Disaster medicine, disaster risk, emergency health, trauma care, and injury). Epidemiology (Not specified). Global Health (General global health topics). Oral and Dental Health (Not specified) Refugee Support (Not specified).

#### Grey literature search

A thorough grey literature search was conducted by MEH using two steps of a WHO-developed mapping framework [18]. Since we are addressing capacity building in LMICs in the MENA region, a review of literature published in non-academic sources is vital to systematically identify such initiatives in this area. The first step included an online search of databases that have hosted GHCB initiatives, trainings, and related activities. As such, filtering was done starting with a general scoping google search for online learning databases that offered global health topics. After assessing several potential databases, we only selected those that allowed us to filter the capacity building initiatives by region so that they meet our set inclusion criteria. Accordingly, we searched for GHCB initiatives using the following databases: UNESCO, International Federation of Medical Students’ Associations, Kaya, Global Health Training Center, and Relief Web. A specific set of keywords was used for the search that included the following terms: “capacity building initiative”, “training programs”, “global health”, “developing countries”, and “low-and-middle income countries”. The keywords were linked with Boolean operators <AND > to limit the breadth of the search and ensure that all concepts were included and < OR > to extend the reach of the search to the entirety of words with similar meaning. The second step included a google web search that aimed to locate capacity-building initiatives not identified by the databases. For the google search, reviewers used the following search term “global health training [country name]”. All relevant links from the first 10 google pages were viewed and assessed for capacity building information that matched the researcher’s criteria. The search for the GHCB initiatives was conducted during a period of 5 months from July 2019 until December 2019. Capacity building initiatives were included in the search if they addressed a global health topic in a LMIC in the MENA, and if they appeared within the first 10 pages of the web-based search.

### Data Collection & Analysis

#### Academic databases

Articles were retrieved by a medical librarian (LH), imported into an Endnote file, and shared with two reviewers (HN and MEH) who conducted the screening process. After a calibration exercise, the two reviewers each screened the titles and abstracts of all studies based on set eligibility criteria. Full texts of all potentially eligible articles were later screened based on the same eligibility criteria. In both phases, a third reviewer (MEK) was assigned to resolve disagreements. Next, one reviewer (HN) extracted the data.

#### Grey literature search

One reviewer (MEH) located the capacity building initiatives from the databases and google searches and extracted the data into an excel sheet.

#### Analysis

Extracted variables from both searches included objective of the initiative, global health topic, target population, country, pedagogic approach, learning modality, outcomes, and funding source. We conducted and reported a descriptive analysis of data gathered from both search strategies. The results illustrated the geographical distribution of initiatives among LMICs in the MENA region, the global health theme of the initiatives, the pedagogic approaches used, the learning modalities, and the target populations.

## Results

### General findings

**Fig. 1:**
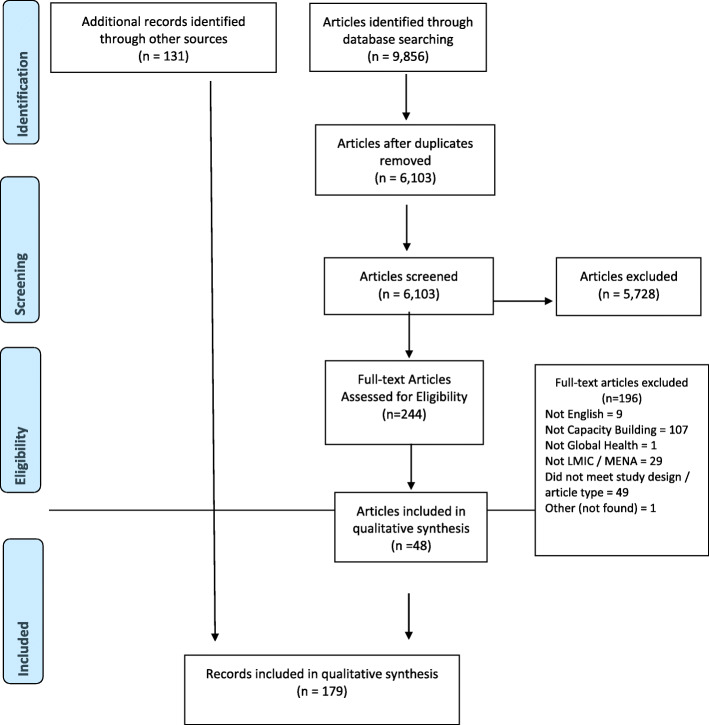
PRISMA Flow Chart

Records included in this review (*n* = 179; see Fig. 1) were mainly from grey sources (see Additional file 2) (*n* = 131, 73.2%), and included governmental and non-governmental reports of GHCB initiatives (see Table 2). With regard to records retrieved from academic sources (26.8% of all records), out of 5972 articles screened (see Fig. 1), 244 were eligible for full-text review, and 48 articles were analysed and had their data extracted (see Table 3). Of all the reviewed records, almost all reported capacity-building initiatives were conducted face-to-face (94.4%), and adopted online (1.7%) or blended (2.2%) learning modalities. Half of the reported GHCB initiatives followed a theory-based (51.4%) pedagogic approach, whereas the rest were interactive (30.6%), mixed theory and practice (11.8%), or were only practical (6.3%). The most frequent target population (see Table 1 for definitions) was professional personnel (57.5%), followed by the general public (18.4%) and community workers (3.9%).

**Table 2:** Summary of Overall Findings

	Grey (*n* = 131)	Academic (*n* = 48)	Total (*n* = 179)
	*N (%)*	*N (%)*	*N (%)*
**Participants**			
Professional	89 (67.9)	14 (29.2)	103 (57.5)
Community	2 (1.5)	5 (10.4)	7 (3.9)
General	8 (6.1)	25 (52.1)	33 (18.4)
Mixed	19 (14.5)	4 (8.3)	23 (12.8)
**Modality**			
Face-to-face	124 (96.9)	45 (93.8)	129 (96.0)
Blended	2 (1.6)	2 (4.2)	4 (2.3)
Online	2 (1.6)	1 (2.1)	3 (1.7)
**Pedagogy**			
Theory	63 (65.6)	11 (22.9)	74 (51.4)
Interactive	17 (17.7)	27 (56.3)	44 (30.6)
Practice & Theory	7 (7.3)	10 (20.8)	17 (11.8)
Practical	9 (9.4)	0 (0)	9 (6.3)
**Global Health Topics**			
Communicable Disease	14 (10.7)	4 (8.3)	18 (10.1)
Child Health	3 (2.3)	0 (0)	3 (1.7)
Disaster and Emergency	15 (11.5)	2 (4.2)	17 (9.5)
Epidemiology	8 (6.1)	2 (4.2)	10 (5.6)
Global Health / General	12 (9.2)	3 (6.3)	15 (8.4)
Health System	15 (11.5)	8 (16.7)	23 (12.9)
Mental Health	18 (13.7)	8 (16.7)	26 (14.5)
Non-Communicable Disease	14 (10.7)	14 (29.2)	28 (15.6)
Oral and Dental Health	0 (0)	2 (4.2)	2 (1.1)
Refugee Support	4 (3.1)	0 (0)	4 (2.2)
Sexual and Reproductive Health	28 (21.4)	5 (10.4)	33 (18.4)
**Countries**			
Algeria	7 (5.3)	0 (0)	7 (3.9)
Djibouti	1 (0.8)	0 (0)	1 (0.6)
Egypt	19 (14.5)	8 (16.7)	27 (15.1)
Iran	3 (2.3)	29 (60.4)	32 (17.9)
Iraq	11 (8.4)	2 (4.2)	13 (7.3)
Jordan	9 (6.9)	1 (2.1)	10 (5.6)
Lebanon	27 (20.6)	3 (6.3)	30 (16.8)
Libya	7 (5.3)	0 (0)	7 (3.9)
Morocco	3 (2.3)	0 (0)	3 (1.7)
Multiple	3 (2.3)	2 (4.2)	5 (2.7)
Syria	5 (3.8)	1 (2.1)	6 (3.4)
Tunisia	10 (7.6)	0 (0)	10 (5.6)
West Bank and Gaza	8 (6.1)	1 (2.1)	9 (5.0)
Yemen	18 (13.7)	1 (2.1)	19 (10.6)

**Table 3:** Bibliography of Academic GHCB Articles among LMICs in the MENA

Country	Topic	Objective	Population	Modality	Pedagogic Approach	Design	Funding
**Egypt**							
Abdel-Aziz et al. (2015) [19]	NCD	To assess maternal knowledge about diarrhea and implement a community-based health and nutrition education messages	General Public	Face-to-face	Interactive	Pre-post Intervention Study	Not mentioned
Abdelazim et al. (2018) [20]	Mental Health	To evaluate the effect of training programs for primary healthcare physicians on the knowledge, attitude, and practice of smoking cessation counselling	Professional Personnel	Face-to-face	Interactive	Pre-post Intervention Study	Not mentioned
Abdelhai et al. (2012) [21]	SRH	To evaluate students’ learning outcomes regarding knowledge acquisition and their opinion towards redesigning a course into an e-learning format	Professional Personnel	Blended	Interactive	Prospective Intervention Study	Swedish Research Link Program; Swedish International Development Agency of the Swedish Research Council in collaboration with the Public Health Department at the Cairo University
Alfaar et al. (2012) [22]	NCD	To Investigate the feasibility of providing clinical pharmacy educational activities through international teleconferencing to improve cancer care in developing countries	Professional Personnel	Online	Theory	Case Study Report	National Cancer Institute; American Lebanese Syrian Associated Charities
El Nouman et al. (2009) [23]	Health System	To improve the performance of healthcare providers, and to promote health among adolescent women	Mixed	Face-to-face	Interactive	Interventional Design	Not mentioned
El-Sayed et al. (2014) [24]	SRH	To evaluate the effectiveness of the WHO course on knowledge and capability of PHC providers, on caretaker’s knowledge and practices, and on children’s’ growth	Mixed	Face-to-face	Theory & Practical	Single-blinded randomized controlled study	WHO and the faculty of Medicine at the Suez Canal University in Egypt
El-Shinawi et al. (2015) [25]	DM	To describe the development and implementation of a trauma training course	Professional Personnel	Face-to-face	Interactive	Case Study Report	National Institutes of Health Fogary International Centre
Roess et al. (2018) [26]	CD	To provide multidisciplinary collaboration and health training for infectious diseases	Professional Personnel	Face-to-face	Interactive	Case Study Report	U.S BEP / U. S & Egypt joint Board
**Iran**							
Ahmadi et al. (2018) [27]	NCD	To compare effect of education delivered by healthcare provider and by peers on self-care behaviours of diabetic patients	General Public	Face-to-face	Interactive	Randomized Control Trial	Rafsanjani University of Medical Sciences
Ardalan et al. (2013) [28]	DM	To evaluate the effectiveness of an intervention on community disaster preparedness in Iran, as delivered by primary healthcare system	General Public	Face-to-face	Theory	Pre-post Intervention Study	National Institute of Health Research, Tehran University of Medical Sciences
Bagherniya et al. (2017) [29]	NCD	To evaluate the impact of a 7-month school-based nutrition education intervention to prevent obesity among adolescent girls	General Public	Face-to-face	Interactive	Cluster Randomized Controlled Trial	Tehran University of Medical Sciences
Didarloo et al. (2016) [30]	NCD	To examine the effect of an educational intervention on behaviour, belief, glycaemic control, and quality of life of women with diabetes.	General Public	Face-to-face	Interactive	Experimental Interventional Study	Tehran University of Medical Sciences
Estebsari et al. (2014) [31]	Global Health	To evaluate the effect on an educational program on aging approach and health promotion behaviour in elderly	General Public	Face-to-face	Interactive	Clinical Trial	Tehran University of Medical Sciences
Ghahremani et al. (2016) [32]	NCD	To examine the effect of educational intervention conducted by volunteers on promoting pap test use among women	Mixed	Face-to-face	Interactive	Quasi-experimental	Research Affairs of Shiraz University of Medical Sciences
Ghahremani et al. (2016) [33]	NCD	To determine the effects of self-care education on performance of breast self-examination among women	General Public	Face-to-face	Theory and practice	Quasi-experimental	Research Affairs of Shiraz University of Medical Sciences
Gholipour et al. (2018) [34]	Health System	To evaluate the district health management training	Professional Personnel	Face-to-face	Theory	Case Study	School of Management and Medical Informatics of Tabriz University of Medial Sciences, Tabriz health Services Management Research Centre and Health Deputy of Tabriz University of Medical Sciences
Javadi et al. (2015) [35]	SRH	To compare the efficacy of lecture and workshop-based trainings on pharmacists’ knowledge of contraception and male sexual dysfunction	Professional Personnel	Face-to-face	Interactive & theory	Randomized Control Trial	Not mentioned
Javanparast et al. (2012) [36]	Health System	To describe the training process of community health workers and how that impacted their performance	Community Workers	Face-to-face	Theory and practice	Qualitative Study	Global Health Research initiative (Canadian institutes of health research, the Canadian international development agency, health Canada, the international development research centre, and the public health agency of Canada)
Jeihooni et al. (2019) [37]	NCD	To assess the effect of education based on the PRECEDE model to promote prostate cancer screening among men	General Public	Face-to-face	Interactive	Quasi-experimental	Not mentioned
Rahbar et al. (2015) [38]	Health System	To describe the of community health workers who deliver health services to health houses of Iran	Community Workers	Face-to-face	Theory and practice	Descriptive / Case Study Report	Not mentioned
Rakhshani et al. (2009) [39]	CD	To enhance knowledge and behaviour of community health workers regarding Malaria	Community Workers	Face-to-face	Theory	Quasi-experimental	WHO / EMRO
Rezaeian et al. (2014) [40]	SRH	To determine the effect of breast cancer screening education on knowledge and beliefs in women using health belief models	General Public	Face-to-face	Interactive	Population-based controlled trial	Not mentioned
Saied-Moallemi et al. (2009) [41]	ODH	To evaluate the effectiveness of a school-based health promotion intervention on pre-adolescent’s oral health	General Public	Face-to-face	Interactive	Randomized community-based trial	Iran Centre for Dental Research (ICDR)
Salamati et al. (2009) [42]	Health System	To assess the effectiveness of a home-based training on disabled people in a rehabilitation program.	General Public	Face-to-face	Theory	Cross-sectional	Not mentioned
Shirani et al. (2019) [43]	Global Health	To evaluate the effect of an educational program on successful aging components in elderly populations	General Public	Face-to-face	Interactive	Randomized Clinical Trial	Isfahan University of Medical Sciences
Orouji et al. (2017) [44]	Mental Health	To assess the effects of educational interventions on smoking cessation behaviour	General Public	Face-to-face	Theory	Randomized Control Trial	Department of health education and promotion, school of public health, Tehran university of medical sciences, Thran Iran
Rabiei et al. (2009) [45]	NCD	To examine the process evaluation of the Isfahan healthy heart program and associated results	General Public	Face-to-face	Interactive	Quasi-experimental	National budget and programming organization; Isfahan cardiovascular research centre
Rezaei et al. (2018) [46]	Mental Health	To examine the effectiveness of psychological intervention in enhancing communication skills of caregivers	General Public	Face-to-face	Theory	Randomized Control Trial	Psychosis research centre, university of social welfare and rehabilitation sciences in Tehran, Iran
Shamsaei et al. (2018) [47]	Mental Health	To study the effect of training interventions relating to stigma on family caregivers of patients with mental illness	General public	Face-to-face	Interactive	Quasi-experimental	Hamdani university of medical sciences, Iran
Siabani et al. (2016) [48]	NCD	To evaluate the effectiveness of a home-based educational strategy delivered through community volunteers to improve self-care of patients with chronic heart failure. This is compared to education delivered by formal health professionals and a control group receiving normal care	General Public	Face-to-face	Interactive	Controlled Trial	Not mentioned
Tavakoly et al. (2018) [49]	NCD	To improve hypertension outcomes and literacy among patients through training of health providers	Mixed	Face-to-face	Interactive	Randomized Control Trial	Mashhad University of Medical Sciences
Termeh et al. (2019) [50]	NCD	To assess the effectiveness of an educational intervention to improve attitudes intention and breast cancer diagnosis among women	General Public	Face-to-face	Interactive	Cluster Randomized Controlled Trial	Not mentioned
Vizeshfar et al. (2019) [51]	Health System	To compare role-pay and lecture-based training on health volunteers’ knowledge	Community Workers	Face-to-face	Interactive	Quasi-experimental	Shiraz University of Medical Sciences
Forghani et al. (2011) [52]	SRH	To compare differences in outcomes between peer-led, and teacher-led HIV prevention material among female high school students in Iran.	General Public	Face-to-face	Interactive	Comparative Study	Not mentioned
Nateghpour et al. (2012) [53]	CD	To examine the difference between regular training versus refresher training courses in the control of malaria	Mixed	Face-to-face	Theory and practice	Case study Report	Centre for Communicable Diseases Control, Ministry of Health and Medical Education, Iran
Omar et al. (2009) [54]	Health System	To evaluate the public health courses and offer recommendations for development of future trainings	Professional Personnel	Face-to-face	Theory and practice	Case Study Report	WHO Khartoum Sudan
Behdjat et al. (2009) [55]	Health System	To examine the value of action research in informing policy-makers regarding healthcare delivery	Community Workers	Face-to-face	Theory and practice	Case Study Report	Not mentioned
**Iraq**							
Mahmood et al. (2018) [56]	Mental Health	To examine the effect of an educational intervention on the knowledge of high school students regarding substance use	General Public	Face-to-face	Interactive	Quasi-experimental	Not mentioned
Murad et al. (2010) [57]	Mental Health	To determine whether trained layperson first responders can improve trauma outcome in a setting where prehospital transit time is long	General Public	Face-to-face	Theory	Non-randomized controlled intervention	Humanitarian grant from the Norwegian Ministry of Foreign Affairs
**Jordan**							
Al Nsour et al. (2018) [58]	Epidemiology	To describe the Jordanian field epidemiology training program, its activities, and its achievements	Professional Personnel	Face-to-face	Theory and practice	Case Study Report	Not mentioned
**Lebanon**							
Arevian et al. (2010) [59]	Mental Health	To train young activists to lead awareness campaigns in Lebanon regarding drug and alcohol abuse and healthy stress management	General Public	Face-to-face	Theory	Case Study Report	Oxfam Canadian Fund for Social Development
Farhood et al. (2010) [60]	Mental Health	To provide mental health training for PHC providers	Professional personnel	Face-to-face	Theory	Interventional Design	WHO office in Lebanon
Karout et al. (2012) [61]	Global Health	To determine the impact of health education intervention on the knowledge, attitudes, and behaviours regarding management of solid wastes of the community	General Public	Face-to-face	Interactive	Randomized semi-controlled intervention study	Not mentioned
**Palestine**							
Ghrayeb et al. (2013) [62]	NCD	To evaluate the impact of a school-based nutrition education intervention on adolescent’s knowledge of nutrition	General Public	Face-to-face	Interactive	Randomized Control Trial	Not mentioned
**Syria**							
Joury et al. (2015) [63]	ODH	To describe the development and evaluation of a program that addressed undergraduates’ knowledge skills and attitudes regarding dental public health. In addition to that, the training aimed to enhance their assessment, and the satisfaction of patients	Professional Personnel	Face-to-face	Theory and practice	Mixed-methods	Not mentioned
**Yemen**							
Al Serouri et al. (2018) [64]	Epidemiology	To describe the Yemen field epidemiology training program, and associated strengths and challenges	Professional Personnel	Face-to-face	Interactive	Case Study Reports	None
**Multiple**							
Mesdaghinia et al. (2013) [65]	CD	To describe the implementation and outcomes of the WHO malaria course	Professional Personnel	Face-to-face	Interactive	Review paper	Not mentioned
Phillimore et al. (2019) [66]	NCD	To describe and evaluate the implementation of a multinational capacity building initiative in the MENA	Professional Personnel	Blended	Theory	Case study report	European Commission

*NCD Non-Communicable Diseases, *CD* Communicable Diseases, *ODH* Oral and Dental Health, *SRH* Sexual and Reproductive Health, *DM* Disaster Management

### GHCB topics

The global health topics that were addressed in the capacity building initiatives included non-communicable diseases, communicable diseases, child health, disaster/emergency preparedness, epidemiology, global health, health system, mental health, oral and dental health, refugee support, and sexual and reproductive health (see Table 1). The most addressed topics were categorized under non-communicable diseases (29.2%), sexual and reproductive health (18.4%) and mental health (14.5%).

### GHCB topics by country

**Fig. 2:**
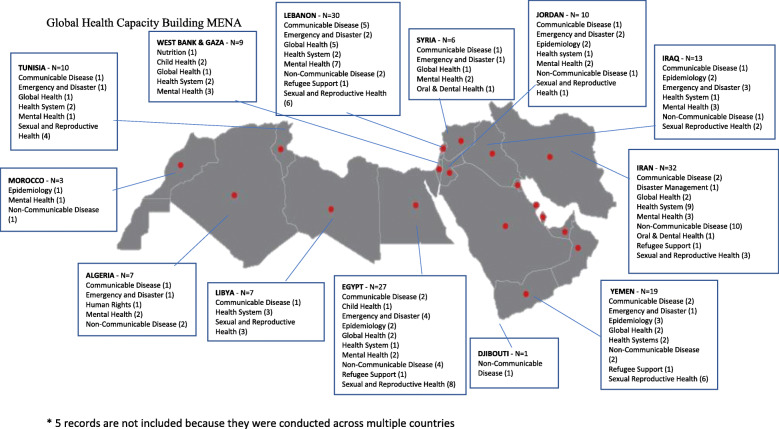
Map of Global Health Capacity Building Initiatives among LMICs in the MENA

The frequency and themes of GHCB initiatives varied by country (see Fig. 2). Iran (*N* = 32), Lebanon (*N* = 30), Egypt (*N* = 27), and Yemen (*N* = 19) reported the highest number of GHCB initiatives. In Iran, non-communicable diseases and health system topics were the most common among the reported GHCB initiatives, whereas in Lebanon GHCB topics mainly targeted mental health, sexual and reproductive health, and communicable diseases. In Yemen, the highest number of reported initiatives addressed sexual and reproductive health, similarly to Egypt who in addition to that, also commonly reported on non-communicable diseases, and emergency and disaster topics.

The least documented GHCB initiatives were derived from West Bank and Gaza (*N* = 9), Algeria (*N* = 7), Libya (*N* = 7), Syria (*N* = 6), Morocco (*N* = 3), and Djibouti (*N* = 1). In Djibouti, only one initiative was reported, and it focused on non-communicable diseases, whereas in Morocco the three reported initiatives targeted mental health, epidemiology, and non-communicable diseases. Initiatives reported from West Bank and Gaza, Algeria, and Syria primarily tackled mental health topics. The highest number of records found from Tunisia addressed sexual and reproductive health topics, and the case was similar in Libya who in addition mostly reported on health system topics. Finally, in Iraq and Jordan, retrieved records mostly targeted emergency and disaster topics, in addition to mental health.

## Discussion

LMICs in the MENA region experience a high burden of disease, and they have limited resources for health education and training [1, 6, 7]. Thus, they have a high need to develop a competent health workforce through GHCB initiatives in order to respond to health challenges. However, very little is known about the topics and approaches of GHCB initiatives being implemented throughout the region. In this systematic review, we summarized GHCB initiatives among LMICs in the MENA region, with a focus on the learning modality, pedagogical approaches, and global health topics. We also matched the documented GHCB topics against the Global Burden of Disease data in order to identify priority areas.

Our findings revealed that over the past decade, all of the reviewed GHCB initiatives among LMICs in the MENA region were conducted face-to-face, with the exception of a handful delivered through online or blended learning modalities. It may be important for LMICs in the MENA region to start adopting innovative learning modalities since these may have strong potential in facilitating the delivery of global health education and training especially in under-served settings with limited resources [67]. For example, there are different reports on digital resources that include online global health courses being available for worldwide use, that have been recommended as effective tools to address the shortage of qualified health workers in LMICs and low-resource settings [68]. Being relevant to some of the health challenges faced in the MENA region, it would be ideal to complement such online courses and distance-based learning platforms with locally-developed, adapted, or contextualized global health material. To that end, more research may be needed to document and evaluate these initiatives along with their effectiveness among LMICs in the MENA region.

Furthermore, our findings showed that theoretical and interactive models were the most commonly used pedagogic approaches in GHCB initiatives, as opposed to practical approaches. Notwithstanding the value of theoretical and interactive approaches, especially those that emphasise active learning, it is also important to complement them with hands-on approaches. Accordingly, it may be important to increase GHCB initiatives that include a practicum or practical component, especially that capacity building in this region is necessary to develop the competency of the workforce to deliver healthcare services.

Overall, professional personnel were the main target groups of the GHCB initiatives, and community workers were the least addressed population. While it is expected that most initiatives would be directed towards professionals, it is crucial for future initiatives to place added attention to community workers. Community health workers play a vital role in healthcare systems, especially those in conflict areas with limited resources, since they can provide less expensive and more tailored services to their communities [69]. Research shows that community workers may be very effective for such purposes, and in many instances, may complement the work of professionals in delivering health-related education to members in some communities largely due to the relationships they build with them [69, 70].

The most commonly addressed GHCB topics among LMICs in the MENA were categorized under non-communicable diseases, sexual and reproductive health, and mental health. Although this is congruent with the overall health needs of the MENA region [1, 71, 72], we have identified some important gaps. First, despite the prevalence of GHCB initiatives that target non-communicable diseases, they were concentrated in Egypt and Iran, and they were under-documented in most other countries. It may be important for other countries such as Lebanon, Jordan, Morocco, Tunisia, and Algeria, to implement and report more efforts regarding GHCB initiatives targeting non-communicable diseases. Second, conflict-related mortalities are among the most common causes of death in the West bank and Gaza, Syria, Libya, Yemen, and Iraq [1, 71], and our results indicate that there is a greater need for emergency and injury-related GHCB in these countries due to their protracted social conflicts. Nevertheless, our findings indicate that mental health GHCB initiatives, which are crucial in war and conflict settings, are commonly reported in some of these countries. Third, although communicable diseases are decreasing overall in the MENA region [1, 71], they still present major concerns in lower-resource settings, and more emphasis should be placed on addressing these topics in countries such as Djibouti.

That said, very few records of GHCB initiatives represented initiatives from Algeria, Djibouti, Libya, Morocco, Syria, and the West Bank and Gaza. These countries, in addition to Jordan, Tunisia, and Yemen, also showed the least academic research activity, given that out of all peer-reviewed articles included in this research, they each had published one or no GHCB study. The majority of initiatives were reported from Egypt, Iran, Lebanon, and Yemen (see Fig. 2). Iran in specific appeared to have the most academic research outputs to disseminate GHCB results. Potentially, as indicated by our findings, this may be related to the availability of local funding for their initiatives, as opposed to the rest of the countries who seemed to rely on international funding from HICs. This may be an important indication supporting the need to prioritize the allocation of resources and funding from local sources to encourage the development, implementation, and dissemination of GHCB initiatives. It is probable that due to the limited publications along with the research gaps in this region [73, 74], many GHCB initiatives may have not been disseminated in the literature and consequently not reported in this review.

## Limitations

Despite the use of two search strategies from grey and academic sources, some records may have still been missed. For example, some initiatives may have not being reported online or disseminated in the literature, especially those in low-resource settings, which may reduce communication among the global health community and which poses a risk for duplication of efforts and inefficiency. Also, while some distance-learning platforms such as Massive Online Open Courses (MOOCs) and others that are available for worldwide use [68, 75], may have reached learners in the MENA region, these were not covered by the scope of our review if they did not explicitly report implementation in a LMIC in the MENA region. Furthermore, some countries in the MENA region may have a lower technical capacity or may be less inclined to allocate resources to publish research outputs. Taken together, these limitations highlight the need to support LMICs in the MENA region to enhance their research production. Additionally, the fact that we only included English records may have limited our range of reviewed records. It is also important to consider that some capacity building records may have not been disseminated under the term “Global Health”, which may have influenced the search and screening process. Finally, although efforts may have been directed to each country’s health needs, we only reviewed records that had a training and/or educational component, and so initiatives such as awareness campaigns and others were not included in this review.

## Conclusion

In light of the escalating global health challenges among LMICs in the MENA region, this systematic review presents the first timely summary and comprehensive analysis of GHCB initiatives being conducted in this setting. Several critical points were identified from this review, such that more GHCB initiatives targeting NCDs and emergency-related topics are needed for most of the reviewed countries. It is also important for this region to increase their adoption of innovative learning modalities and practical and hands-on approaches, and to target more community health workers. Finally, it may be essential for countries to prioritize and mobilise resources and local funding to increase the development, implementation, and dissemination of GHCB initiatives.

## Supplementary information

**Additional file 1.** Search Strategy.

**Additional file 2.** Bibliography of Grey Literature GHCB sources among LMICs in the MENA

## Data Availability

The datasets used and/or analysed during the current study are available from the corresponding author on reasonable request.
