# Supplementary material for: A systematic review of global health capacity building initiatives in low-to middle-income countries in the Middle East and North Africa region
Source: Global Health. 2020 Jul 3;16:56. doi: 10.1186/s12992-020-00585-0 (PMC7333284; doi:10.1186/s12992-020-00585-0)
Supplement: Supplementary file 1 — Additional file 1. Search Strategy. [file 12992_2020_585_MOESM1_ESM.docx]

**Appendix I.**

**Search Strategy.**

Capacity Building[Mesh:noexp] OR healthcare literacy[tw] OR health literacy[tw] OR program literacy[tw] OR teaching[tw] OR teachings[tw] OR teachback[tw] OR teach-back[tw] OR course[tw] OR courses[tw] OR webinar[tw] OR webinars[tw] OR e-learning[tw] OR elearning[tw] OR learning[tw] OR learnings[tw] OR online-learning[tw] OR education[tw] OR educational material*[tw] OR healthcare information[tw] OR health information[tw] OR health promotion[tw] OR healthcare promotion[tw] OR health programs[tw] OR health program[tw] OR health programme[tw] OR health programmes[tw] OR healthcare programme[tw] OR healthcare programmes[tw] OR healthcare programs[tw] OR healthcare program[tw] OR program literacy[tw] OR programme literacy[tw] OR health literacy[tw] OR healthcare literacy[tw] OR health promotion[tw] OR health campaign[tw] OR health campaigns[tw] OR health awareness[tw] OR healthcare promotion[tw] OR healthcare campaign[tw] OR healthcare campaigns[tw] OR healthcare awareness[tw] OR wellness program[tw] OR wellness programs[tw] OR wellness programme[tw] OR wellness programmes[tw] OR wellness campaign[tw] OR wellness campaigns[tw] OR smoking campaign[tw] OR smoking campaigns[tw] OR smoking awareness[tw] OR smoking program[tw] OR smoking programs[tw] OR smoking programme[tw] OR smoking programmes[tw] OR anti-smoking campaign[tw] OR anti-smoking campaigns[tw] OR anti-smoking awareness[tw] OR anti-smoking program[tw] OR anti-smoking programs[tw] OR anti-smoking programme[tw] OR anti-smoking programmes[tw] OR antismoking campaign[tw] OR antismoking campaigns[tw] OR antismoking awareness[tw] OR antismoking program[tw] OR antismoking programs[tw] OR antismoking programme[tw] OR antismoking programmes[tw] OR health fair[tw] OR health fairs[tw] OR healthcare fair[tw] OR healthcare fairs[tw] or MOOCs[tw] OR training[tw] OR trainers[tw] OR trainer[tw] OR ToT[tw] OR Pedagogic approach[tw] OR Pedagogical approach[tw] OR webcast[tw] OR webcasts[tw] OR tutorial[tw] OR tutorials[tw] OR discussion forum[tw] OR discussion board[tw] OR videoconference[tw] OR videoconferences[tw] OR videoconferencing[tw] OR video-conference[tw] OR video-conferences[tw] OR video-conferencing[tw] OR moodle[tw] OR blackboard[tw] OR Teaching Materials[Mesh:noexp] OR teaching material*[tw] OR Program Development[Mesh:noexp] OR program development[tw] OR programme development[tw] OR progrmame description[tw] OR program description[tw] OR “Fellowships and Scholarships”[ Mesh:noexp] OR Fellowship[tw] OR Fellowships[tw] OR scholarship[tw] OR scholarshipprogram[tw] OR scholarships[tw] OR global intervention[tw] OR global interventions[tw] OR global initiative [tw] OR global initiatives[tw]

OR "access to information"[MeSH:noexp] OR Access to Information[text] OR FOIA Requests OR open access[text] OR information Dissemination [text] OR "Information Dissemination"[Mesh:NoExp] OR data Dissemination*[text] OR data distribution[text] OR data distributions[text] OR data sharing*[text] OR information Dissemination*[text] OR "Problem Solving"[Mesh:NoExp] OR problem solving[text] OR problem solvings[text]

AND

Global Health[Mesh:noexp] OR Public Health[Mesh:noexp] OR world health [tw] OR worldwide health[tw] OR global health[tw] OR international health[tw] OR national health[tw] OR public health[tw] OR community health[tw] OR information distribution[text] OR information distributions[text] OR information sharing*[text]

AND

africa, northern[mesh] OR middle east[mesh:noexp] OR iraq[mesh] OR jordan[mesh] OR iran[mesh] OR lebanon[mesh] OR syria[mesh] OR yemen[mesh] OR algeria*[tw] OR djibouti[mesh] OR egypt*[tw] OR iraq*[tw] OR jordan*[tw] OR iran*[tw] OR lebanon[tw] OR lebanese[tw] OR libanaise[tw] OR yemen*[tw] OR aden[tw] OR sanaa[tw] OR libya*[tw] OR morocco[tw] OR moroccan*[tw] OR palestin*[tw] OR gaza[tw] OR ghazza[tw] OR ghaza[tw] OR west-bank[tw] OR syria*[tw] OR tunis*[tw] OR ifni[tw] OR trucial state*[tw] OR north africa*[tw] OR northern Africa*[tw] OR MENA[tw] OR EMRO[tw] OR middle east*[tw] OR east mediterranean[tw] OR eastern mediterranean[tw] OR near east*[tw] OR orient[tw] OR djibouti*[tw] OR levant[tw]
