# Supplementary material for: A systematic review of global health capacity building initiatives in low-to middle-income countries in the Middle East and North Africa region
Source: Global Health. 2020 Jul 3;16:56. doi: 10.1186/s12992-020-00585-0 (PMC7333284; doi:10.1186/s12992-020-00585-0)
Supplement: Supplementary file 2 — Additional file 2. Bibliography of Grey Literature GHCB sources among LMICs in the MENA [file 12992_2020_585_MOESM2_ESM.docx]

**Table 4**. Bibliography of Grey Literature GHCB sources among LMICs in the MENA

| **Title of Initiative** | **Implementation Organization** | **Topic** | **Objective** | **Population** | **Modality** | **Pedagogic Approach** | **Funding Source** |
| --- | --- | --- | --- | --- | --- | --- | --- |
| **Algeria** | | | | | | | |
| Their Fight Story | IFMSA (1) | MH | To provide attendees with a new perspective on cancer and provide training and awareness of relevant MHPSS skills necessary when working with cancer patients | General Public | Face-to-Face Learning | Interactive | Not mentioned |
| What do you know about human rights? | IFMSA (2) | GH | To raise awareness of the rights of patients and doctors, the Universal Declaration of Human Rights, and tips to avoid violence in hospital environments & respect rights | Professional Personnel | Face-to-Face Learning | Interactive | Not mentioned |
| أنت تهمينا ... افحصي وطمنينا  You are important for us ... Examine and reassure us | IFMSA (3) | NCD | To teach university women how to conduct breast self-examination and encourage mammogram screening | Professional Personnel | Face-to-Face Learning | Theory | Not mentioned |
| Medicine is not candy | IFMSA (4) | CM | To educate the public of dangers of self-medication and AMR and provide practitioners/pharmacists with best-practices when informing patients about medication use | Mixed | Face-to-Face Learning | Not mentioned | Not mentioned |
| Ma souffrance n'est pas drôle #Manich_Mahboul (My suffering is not funny #Im_Not_Crazy) | IFMSA (5) | MH | To raise awareness of mental illnesses and combat discrimination/stigma by promoting respect, with a focus on depression, schizophrenia, and health & human rights | Mixed | Face-to-Face Learning | Theory & Practice | Not mentioned |
| Disaster Risk Reduction for Resilience | UN Office for Disaster Risk Reduction (6) | DE | To build teams’ capacity to expand support to Algerian partners as part of Strategic Cooperation Framework; To provide training on how to contribute to the local rollout of initiatives to make cities more resilient to disasters and to build safer schools/hospitals | Professional Personnel | Face-to-Face Learning | Theory | Not mentioned |
| Healthcare Professional Training (Diabetes) | Novo Nordisk (7) | NCD | To enhance knowledge about medical and behavioural interventions | Professional Personnel | Face-to-Face Learning | Interactive | Not mentioned |
| **Djibouti** | | | | | | | |
| Capacity building exercises to tackle malnutrition | Action Against Hunger & MOH (8) | NCD | To tackle malnutrition across Djibouti | Professional Personnel | Face-to-Face Learning | Not mentioned | European Commission Directorate General for Humanitarian Aid and Civil Protection (ECHO) |
| **Egypt** | | | | | | | |
| No slum areas (NOSA) | IFMSA (9) | GH | To enhance the ability of families in fragile areas to better manage their health & well-being via volunteers who provide health services and by raising awareness among medical students | Mixed | Face-to-Face Learning | Practice | Not mentioned |
| Breast Cancer "Pink October' | IFMSA (10) | NCD | To spread awareness of Breast Cancer among medical students through workshops during 2016 & 2017 | Mixed | Face-to-Face Learning | Not mentioned | Not mentioned |
| World AIDS Campaign | IFMSA (11) | SRH | To train trainers and 20 medical students within each Local Committee on AIDS by November 2017 | Mixed | Face-to-Face Learning | Not mentioned | Not mentioned |
| Child Abuse Prevention | IFMSA (12) | CH | To raise children and parents’ awareness of child abuse via theatrical scenarios, music, and play | General Public | Face-to-Face Learning | Theory & Practice | Not mentioned |
| Anti-Microbial Resistance | IFMSA (13) | CD | To advocate for adding AMR to the curricula of medical and paramedical colleges by conducting/publishing research, launching campaigns, & creating national TOT | Professional Personnel | Face-to-Face Learning | Not mentioned | Not mentioned |
| Adolescent's Health | IFMSA (14) | SRH | To conduct training sessions on sexual and reproductive health among students, and provide sessions to teachers and caregivers on working/teaching youth about SRH | Mixed | Face-to-Face Learning | Not mentioned | Not mentioned |
| MSSA 11th Annual Conference - Disaster medicine in Egypt | IFMSA (15) | DE | To spread knowledge of Disaster Risk Reduction (DRR), train medical students on disaster management and disaster medicine, and raise awareness of DRR measures and disaster medicine to the public via social media | Mixed | Face-to-Face Learning | Not mentioned | Not mentioned |
| Home-mates | IFMSA (16) | GH | To provide families with access to hospitals and basic services through medical convoys as well as access to clean water, and inform them of common diseases & importance of sanitation through campaigns; To motivate medical students and the public to participant in SDGs via online campaigns and local workshops | Professional Personnel | Face-to-Face Learning | Not mentioned | Not mentioned |
| Anti-Sexual Harassment | IFMSA (17) | SRH | To conduct an awareness workshop for female medical students about sexual harassment and PTSD; To increase physical fitness and self-defence abilities of female students from Mansoura University | Professional Personnel | Face-to-Face Learning | Not mentioned | Not mentioned |
| Anti-FGM | IFMSA (18) | SRH | To build a core team of IFMSA-Egypt members to hold anti-FGM campaigns targeting medical students and to raise awareness of the public towards FGM through health education programs in PHC units | Mixed | Face-to-Face Learning | Not mentioned | Not mentioned |
| Mr. & Ms. Breastestis | IFMSA (19) | NCD | To raise awareness of breast and testicular health via physical campaigns in urban and rural areas, distribution of flyers, online campaigns for breast and testicular cancer, providing portable mammogram machines, and fundraisers to support local oncology centers | Mixed | Face-to-Face Learning | Not mentioned | Not mentioned |
| Mental Health | IFMSA (20) | MH | To raise awareness and train medical students about mental illness and how to end stigma | Professional Personnel | Face-to-Face Learning | Not mentioned | Not mentioned |
| Egypt Field Epidemiology Training Program | Training Programs in Epidemiology and Public Health Interventions Network (21) | EPI | To train public health workers to provide essential epidemiologic services | Professional Personnel | Face-to-Face Learning | Practice | Not mentioned |
| Strengthening Egypt's family Planning Program | USAID (22) | SRH | To provide technical assistance and training for Ministry of Health and Population staff to improve the quality of family planning services to stem growth of fertility rate | Professional Personnel | Face-to-Face Learning | Theory | Not mentioned |
| Egypt’s Ministry of Health and Population Completes Fourth Subnational Emergency Rapid Response Teams Training Workshop | EMPHNET (23) | DE | To enhance participants' skills and capacities to respond to outbreaks and other health emergencies | Professional Personnel | Face-to-Face Learning | Theory | GHD/EMPHNET |
| GHD/EMPHNET Supports Capacity Building for Rapid Response Teams in Egypt | EMPHNET (24) | DE | To enhance the capacities of RRT members to respond to outbreaks and other health emergencies | Professional Personnel | Face-to-Face Learning | Theory | GHD/EMPHNET |
| Egypt Ministry of Health and Population Conducts Workshop Two of Public Health Empowerment Program – Basic Field Epidemiology (PHEP-BFE) | EMPHNET (25) | EPI | To train the public health workforce in the principles and practices of field epidemiology | Professional Personnel | Face-to-Face Learning | Theory | GHD/EMPHNET |
| Egypt ICRC and LAS hold training on sexual violence in armed conflicts | ICRC and League of Arab States (26) | SRH | To provide information on various topics related to sexual violence in armed conflict | Professional Personnel | Face-to-Face Learning | Theory | Not mentioned |
| International Refugee Law | American University in Cairo (27) | Refugee Support | To teach participants on the basic features of International Refugee Law through the lens of the 1951 Refugee Convention | Professional Personnel | Face-to-Face Learning | Interactive | Not mentioned |
| **Iran** | | | | | | | |
| West Azerbaijan Project |  | HS | To expand medical/health services by establishing a comprehensive health delivery system and training HCPs | Professional Personnel | Face-to-Face Learning | Theory | Not mentioned |
| Training | The National Public Health Management Center (28) | HS | To develop the capacity of trainees to apply the art of management to the health systems of organizations | Professional Personnel | Face-to-Face Learning | Theory | Not mentioned |
| Migration and Development Training | IOM (29) | Refugee Support | Enhance participants’ understanding of how to mainstream migration into development planning | Professional Personnel | Face-to-Face Learning | Theory | Government of Japan |
| **Iraq** | | | | | | | |
| Sexually Transmitted Diseases including AIDS Campaign | IFMSA (30) | SRH | To deliver appropriate medical attention and follow-up to patients living with HIV/AIDS and other STI’s | Mixed | Face-to-Face Learning | Not mentioned | Not mentioned |
| The Anti-Smoking Project | IFMSA (31) | NCD | To decrease the incidence of smokers and passive smokers and highlight the deleterious effects of tobacco as a risk factor for NCDs in Jordan | Professional Personnel | Face-to-Face Learning | Not mentioned | Not mentioned |
| Iraq Field Epidemiology Training Program | Training Programs in Epidemiology and Public Health Interventions Network (32) | EPI | To reduce the burden of major public health problems by training a cadre of public health workers who can provide essential epidemiologic services to the country. | Professional Personnel | Face-to-Face Learning | Practice | Not mentioned |
| Training course on gender-based violence for medical and health staff working in the Medical City Department | Ministry of Health (33) | SRH | To raise awareness about GBV at global and regional levels and provide HCPs with tools to address GBV issues on the ground | Professional Personnel | Face-to-Face Learning | Theory | Not mentioned |
| Preparatory workshop on the project of strengthening capacities in the field of applied epidemiology | Ministry of Health and Organization for the prevention of risk (34) | EPI | To develop a workforce in the field of applied epidemiology from current medical and health staff | N/A | Face-to-Face Learning | Theory | Not mentioned |
| Strengthening Global Capacity for Emergency Health | UNFPA, Global Health Cluster, Avenir Analytics (35) | DE | To clarify and address the imbalance between emergency health response capacity supply and demand | Professional Personnel | Face-to-Face Learning | Theory | Not mentioned |
| Training courses on biosafety, biosecurity, and diagnostic skills | Communicable Disease Center (36) | Health Safety | To conduct continuing education trainings following the CRDF Global Iraq Fellowship Program | Professional Personnel | Face-to-Face Learning | Theory | Not mentioned |
| ATLS/PALS Training | Medical college of Wisconsin Office of Global Health (37) | DE | To train first year graduates from the University of Dohuk in battle torn areas and assist with surgical cases among the local refugee population | Professional Personnel | Face-to-Face Learning | Theory | Not mentioned |
| First responder training | International Medical Corps (38) | DE | To provide residents with skills and tools to administer effective and essential care at the scene of an emergency | Professional Personnel | Face-to-Face Learning | Theory & Practice | EU Humanitarian budget |
| Training | International Medical Corps (39) | CD | To expand knowledge regarding common diseases, educational messages, and simple case definitions. | Mixed | Face-to-Face Learning | Theory | Not mentioned |
| mhGAP training | International Medical Corps (40) | MH | To provide mhGAP and Psychological First Aid (PFA) training. | Professional Personnel | Face-to-Face Learning | Theory | Not mentioned |
| **Jordan** | | | | | | | |
| It’s on us (NCD) | IFMSA (41) | NCD | To raise awareness of NCDs and healthy lives and provide free screenings for the public; to educate medical students of NCDs and task them with spreading awareness of healthy lifestyles | Mixed | Face-to-Face Learning | Not mentioned | Not mentioned |
| Here I Am: National Mental Health Project | IFMSA (42) | MH | To address stigma surrounding mental health through awareness events, workshops, training sessions | General Public | Face-to-Face Learning | Not mentioned | Not mentioned |
| Jordan Field Epidemiology Training Program | Training Programs in Epidemiology and Public Health Interventions Network (43) | EPI | To reduce the burden of major public health problems by training a cadre of public health workers who can provide essential epidemiologic services to the country. | Professional Personnel | Face-to-Face Learning | Practice | Not mentioned |
| Theoretical and practical training | International Medical Corps (44) | SRH | To provide ongoing theoretical and practical training on child protection and gender-based violence management and referral. | Professional Personnel | Face-to-Face Learning | Theory & Practice | Not mentioned |
| MHPSS training | International Medical Corps (45) | MH | To train Ministry of Health staff and other HCPs in MHPSS to enhance resilience and improve capacities of existing service providers while increasing access for refugees and host communities. | Professional Personnel | Face-to-Face Learning | Theory | Not mentioned |
| EMPHNET Conducts Workshop to Build a Pool of Potential Consultants for Strengthening Routine Immunization at the Regional Level | EMPHNET, CDC, WHO, UNICEF, IFRC, and ministries of health in the region (46) | CD | To strengthen routine immunization in the Eastern Mediterranean Region (EMR) |  | Face-to-Face Learning | Interactive | Not mentioned |
| Pre-Conference Workshop on “Contemporary Issues on Public Health in Emergency | EMPHNET (47) | DE | To familiarize public health professionals in the EMR with knowledge of public health in emergencies and to enable them to effectively respond and manage health humanitarian crises. | Professional Personnel | Face-to-Face Learning | Interactive | Not mentioned |
| Fogarty project provided emergency trauma training to Iraqis | NIH Fogarty International Center (48) | DE | To aid the Kurdish Health Ministry to establish a formal medical emergency plan, including plans for rescue, decontamination, triage, stabilization, evacuation, and treatment | Professional Personnel | Face-to-Face Learning | Theory | Fogarty |
| Safe and Secure Approaches in Field Environments (SSAFE) Training Course | UN Department of Safety and Security (49) | HS | To raise participants’ awareness of prevailing threat in their operational environments and equip them with pertinent knowledge and skills required to prevent and respond to various security incidents that they might encounter in the field | Professional Personnel | Face-to-Face Learning | Theory & Practice | Not mentioned |
| **Lebanon** | | | | | | | |
| Orphanage health day- second addition | IFMSA (50) | GH | To offer medical check-ups for orphans;  To enhance health awareness among children via games;  To provide youth with knowledge of health challenges;  To equip volunteers with communication and medical skills to care for orphans;  To provide project management and impact measurement skills to select members;  To implement this activity annually and to an additional orphanage to ground the concept of sustainability and continuous impact in the work of SCOPH. | Mixed | Face-to-Face Learning | Not mentioned | Not mentioned |
| Food Safety Campaign | IFMSA (51) | CD | To equip medical students with skills in the field of food safety via training by the ministry and SCOPH | Mixed | Face-to-Face Learning | Not mentioned | Training covered by the ministry of economy and trade |
| International Training on Disaster Medicine | IFMSA (52) | DE | To provide medical students with basic knowledge of Disaster Medicine. To provide a platform to analyse medical ethical dilemmas and for trainers to train medical students | Professional Personnel | Face-to-Face Learning | Interactive | Not mentioned |
| Training on the concept of srhr, child marriage and working with adolescents | ABAAD (53) | SRH | To provide service providers with knowledge of sexual and reproductive health and rights (SRHR) and provide them with interactive tools to utilize in the field | Professional Personnel | Face-to-Face Learning | Not mentioned | Not mentioned |
| Comprehensive training on gender based violence case management in emergencies | ABAAD (54) | SRH | To equip social workers and case managers with knowledge of GBV case management in emergencies, emphasis on the women’s human rights framework and in line with internationally-recognized standards of good practice, and to provide them with IEC materials and Tools for community education and outreach on GBViE | Professional Personnel | Face-to-Face Learning | Not mentioned | Not mentioned |
| Training on how to organize support groups for women survivors of GBV using a survivor-centered approach | ABAAD & HIVOS (55) | SRH | To reinforce the capacity of participants in planning and facilitating support group sessions with women survivors of GBV using a survivor-centered approach. | Professional Personnel | Face-to-Face Learning | Not mentioned | Not mentioned |
| Training on how to engage front-line workers in combating child marriage | ABAAD (56) | SRH | To introduce participants to the inter-linkages between child marriage and GBV against girls and provide them with interactive tools and exercises to catalyse discussions on the topic with adolescent girls and their families. | Professional Personnel | Face-to-Face Learning | Not mentioned | Not mentioned |
| LGBT Mental Health Training Program | Lebanese Medical Association for Sexual Health (57) | MH | To train mental health and psychosocial support professionals on best practices when working with LGBT individuals | Professional Personnel | Face-to-Face Learning | Theory | U.S AID |
| Slia - Young People and Drugs: A new participator approach for Lebanon | Skoun, Lebanese Addiction Center, and SIDC (58) | MH | To improve the capacity of civil society organizations and governmental bodies to address problems related to drug abuse among young people in Lebanon | Professional Personnel | Face-to-Face Learning | Theory | Social justice Lebanon (EU) |
| Building the capacity of emergency room staff on managing psychiatric emergencies | National mental health program in Lebanon (59) | MH | To build the capacity of emergency room staff on managing psychiatric emergencies | Professional Personnel | Face-to-Face Learning | Theory | WHO |
| Integration of non-communicable diseases services within primary healthcare | MoPH (60) | NCD | To integrate the non-communicable disease program based on the WHO model within primary healthcare centres in Lebanon | Professional Personnel | Face-to-Face Learning | Theory | Not mentioned |
| Promoting psychosocial and mental health care through community-based educational workshops for teachers and parents | AUB School of Nursing (61) | MH | To assess the efficacy of a school-based mental health intervention provided to teachers and parents of students from different school levels | General Public | Face-to-Face Learning | Theory | Not mentioned |
| Training course on surveillance and response system for communicable diseases in health centres and dispensaries | MoPH (62) | CD | To alleviate the impact of the Syrian crisis on Lebanon by: strengthening the public healthcare system’s capacity to manage communicable diseases, providing quality primary health care services, and ensuring adequate provision of vaccines and medications throughout Lebanon | N/A | Face-to-Face Learning | Theory | European Union |
| Service delivery guidelines for reproductive health | MoPH (63) | SRH | To improve the knowledge and skills of service providers to ensure the provision of reproductive health services that meet women, men's and youth's needs in Lebanon. | Professional Personnel | Face-to-Face Learning | Theory | Not mentioned |
| NCD Trainings | MoPH (64) | NCD | To ensure the proper implementation of NCD programs in primary health care services, and the proper use of program tools | Professional Personnel | Face-to-Face Learning | Theory | Not mentioned |
| Training workshop for nurses with order of nurses in Lebanon | MoPH (65) | CD | To raise awareness and educate of the primary role of the nurse through health care, patient education, public awareness and guidance. | Professional Personnel | Face-to-Face Learning | Theory | Not mentioned |
| Proficiency Testing Training Project under the AMR Program | MoPH (66) | CD | To train laboratories on the quality of tests on microbes and antibiotic-resistant germs, allowing the development of a scientific vision based on effective challenges. |  | Face-to-Face Learning | Theory | WHO |
| Regional training to strengthen response to humanitarian needs held in Beirut | ICRC (67) | Refugee Support | To strengthen the capacity of the leaders of National Societies in order to improve their response to the humanitarian needs in their respective countries. | Professional Personnel | Face-to-Face Learning | Theory | Not mentioned |
| Case focused psychosocial work and general activities to support MHPSS | International Medical Corps (68) | MH | To provide MHPSS activities and service provision |  | Face-to-Face Learning | Theory | Not mentioned |
| Field Research Team members training | Médecins du Monde (69) | GH | To offer intensive technical training for field team members covering data and sample collection and storage, as well as generic skills such as communication with adults and children, ethics and personal conduct and security. | Professional Personnel | Face-to-Face Learning | Practice | Not mentioned |
| Nutrition in Emergency Course | AUB (70) | CD |  | Professional Personnel | Face-to-Face Learning | Interactive | Not mentioned |
| Community health trainings | International Medical Corps (71) | GH | To provide in-depth training and field coaching regarding a variety of health education topics, including infectious diseases, non-communicable diseases and other chronic illnesses, infant and young child feeding nutrition for the life cycle, immunization, newborn care, antenatal care, personal hygiene and sexually transmitted diseases. | General Public | Face-to-Face Learning | Theory | Not mentioned |
| Workshop on Responding to Refugees’ Health Needs | AUB, in collaboration with the Columbia University Mailman School of Public Health (72) | HS | To discuss the research’s key findings of challenges facing the region’s health systems in relation to service provision and barriers to service access. Also to present findings on the coverage and quality of health services, and the lessons learned from previous experiences in health response, focusing on the case of responding to HIV among refugees in the region. | Professional Personnel | Face-to-Face Learning | Interactive | Not mentioned |
| Mobile University for Health Women’s Health Certificate | Global Health Institute (73) | SRH | To enable participants to apprehend women’s health and describe its significance in the health field especially in vulnerable settings | General Public | Blended Learning | Theory & Practice | Open Society Foundation |
| Global Burden of Disease Training | Global Health Institute and Health Metrics and Evaluation (IHME) (74) | GH | To introduce participants to the foundations, principles and applications of the Global Burden of Disease Study (GBD), including real-world examples of how countries are utilizing it for decision making and hands-on exercises to familiarize participants with the many resources available. | Professional Personnel | Face-to-Face Learning | Theory | Not mentioned |
| Hostile Environment Surgical Training Course | GHI Academy and David Nott Foundation (75) | DE | To teache the essential surgical decision-making strategies and techniques for surgeons working in austere conditions. | Professional Personnel | Face-to-Face Learning | Interactive | Not mentioned |
| Global Health Diplomacy Workshop | GHI Academy and Graduate Institute of Geneva Global Health Centre (76) | GH | To understand key concepts in global health and global health diplomacy, gain insights into current global health challenges and complexities in Low and Middle Income countries, explore the role of global health diplomacy in enhancing social cohesion among refugees and host communities, identify health diplomacy challenges in conflict settings, determine the elements of an intersectoral collaborative approach for setting the agenda for NCDs, improve negotiations skills through case studies discussed in working groups | Professional Personnel | Face-to-Face Learning | Interactive | Not mentioned |
| **Libya** | | | | | | | |
| Training course on the use of DHIS2 in several municipalities in Libya | MOH (77) | HS | To train statistical staff in health facilities on the use of district health information systems | Professional Personnel | Face-to-Face Learning | Theory | Not mentioned |
| Training of trainers on district health information system DHIS2 | MOH (78) | HS | To enrich the capacity of health information staff at facility and municipality level on data entry, analysis, reporting of disease burden, and utilization data; also to enhance their teaching and supervisory skills | Professional Personnel | Face-to-Face Learning | Theory | Not mentioned |
| Healthcare Training in Libya | International Medical Corps (79) | SRH | To train participants in reproductive health and emergency obstetrics. | Professional Personnel | Face-to-Face Learning | Theory | European Union Civil Protection and Humanitarian Aid |
| RRT Training of Trainers Workshop | GHD/EMPHNET and Libya National Communicable Disease Center (80) | DE | To provide a refresher on RRT topics, especially in relevance to the Libyan context. Topics covered included the composition of RRT, the International Health Regulations (IHR), surveillance systems, outbreak investigation and control measures, risk assessment and communication, infection control and prevention, biosafety and biosecurity, among other topics. | Professional Personnel | Face-to-Face Learning | Theory | Not mentioned |
| Training in Reproductive Health and Mental Health and Psychosocial Support | International Medical Corps (81) | SRH | To deliver specialized training on reproductive health and mental health and psychosocial support | Professional Personnel | Not mentioned | Not mentioned | Not mentioned |
| Capacity Building to diagnosis and case management for leishmaniosis using WHO and National guidelines | WHO (82) | CD | N/A | N/A | Not mentioned | Not mentioned | Not mentioned |
| Action for Mental Health Assistance | WHO (83) | SRH | To enhance access to mental health care services (destigmatisation of mental health disorders, provision of community based psychosocial interventions), capacity building of health professionals (specialized and non-specialized) and offer of a specialized education curriculum in mental health. | Professional Personnel | Face-to-Face Learning | Not mentioned | European union |
| Training of Trainers on District Health Information System (DHIS-2) | Ministry of Health, WHO, UNICEF and IOM (84) | HS | To enrich the capacity of MOH staff on data entry, analysis and reporting of disease burden and service utilization data but will also enhance their teaching and supervisory skills. | Professional Personnel | Face-to-Face Learning | Theory | European union |
| **Morocco** | | | | | | | |
| Fighting addiction with knowledge | IFMSA (85) | MH | To increase knowledge of students about the dangers of tobacco and drugs  (training the medical students to disseminate knowledge to high school students) | General Public | Face-to-Face Learning | Not mentioned | Not mentioned |
| Morocco Field Epidemiology Training Program | Training Programs in Epidemiology and Public Health Interventions Network (86) | EPI | To reduce the burden of major public health problems by training a cadre of public health workers who can provide essential epidemiologic services to the country. | Professional Personnel | Face-to-Face Learning | Practice | Not mentioned |
| Chevron’s health care training | Chevron (87) | NCD | To train doctors and specialists in blood diseases and cancer | Professional Personnel | Face-to-Face Learning | Theory | Not mentioned |
| **Syria** | | | | | | | |
| Training course on international health regulations | MOH (88) | GH | Variation of topics discussed in the training course such as health regulation exercises, epidemiology of emerging disease, specific topics of cholera and Ebola… | N/A | Face-to-Face Learning | Theory | Not mentioned |
| ICRC trains 57 national society volunteers in water and sanitation | ICRC (89) | CD | To train the volunteers on how to handle water supply systems in emergency crises. | General Public | Face-to-Face Learning | Practice | Not mentioned |
| Case focused psychosocial work and general activities to support MHPSS | International Medical Corps (90) | MH | To provide MHPSS activities and service provision |  | Face-to-Face Learning | Theory | Not mentioned |
| EWARN (early warning and response network) surveillance system training | CDC (91) | DE | To conduct training to Syrian staff in Southern Syria on the early warning and response network system | Professional Personnel | Face-to-Face Learning | Theory | Not mentioned |
| Training in MHPSS | International Medical Corps (92) | MH | To train local health workers in mental health and psychosocial support (MHPSS) as well as protection services, strengthening the area’s capacity for resilience. | Community Workers | Face-to-Face Learning | Theory | Not mentioned |
| **Tunisia** | | | | | | | |
| AntiMicrobial Resistance Program | IFMSA (93) | CD | To educate medical students and the general public on the mechanisms, epidemiology, and impact of Antimicrobial Resistance (AMR). To educate medical students and other health professionals on the relationship between veterinary practices, the food chain and AMR in human communicable diseases. To equip medical students with the tools to critically examine antimicrobial strategies locally, regionally, nationally, and internationally, and advocate for changes to antimicrobial stewardship practices that increase AMR | Mixed | Face-to-Face Learning | Interactive | Not mentioned |
| SCORA Generation | IFMSA (94) | SRH | To provide young people with the tools to make reasonable decisions and build healthy relationships To help youth delay onset of sexual activity, reduce the frequency of unprotected sexual activities and increase condom and contraceptive use. To foster respect for human rights and diversity as a way to reduce stigma and discrimination To communicate a positive, life-cycle approach to sexuality To help strengthen communication skills and decision-making abilities through including participatory teaching methods To provide honest, effective sex education – not ineffective, shame-based abstinence-only programs | General Public | Face-to-Face Learning | Interactive | Not mentioned |
| Mental Health Projects | IFMSA (95) | MH | To educate medical students on mental health issues (training, workshops) | Professional Personnel | Face-to-Face Learning | Not mentioned | Not mentioned |
| International course on maternal and neonatal health focused on reducing maternal mortality | MOH (96) | SRH | To teach about medical and educational services for safe motherhood through theoretical courses and practical training | Professional Personnel | Face-to-Face Learning | Theory & Practice | Not mentioned |
| International training session in reproductive health program management | MOH (97) | SRH | To reduce the maternal and infant mortality rate and strengthen the perinatal health coverage in this region. | Professional Personnel | Face-to-Face Learning | Not mentioned | Not mentioned |
| Introduction to Global Health | Organisation Mondiale de la Sante Tunisie (98) | GH | To provide an understanding of global burden of diseases, health systems, universal health coverage and global health stakeholders. | N/A | Face-to-Face Learning | Interactive | Not mentioned |
| Third country training for youth reproductive health | Japan International Cooperation Agency (99) | SRH | To assist in improving women’s health care management, population control and HIV/AIDS prevention. | Professional Personnel | Face-to-Face Learning | Theory & Practice | Not mentioned |
| NEBOSH Training Award in Health and Safety at Work | National Examination Board in Occupational Safety and Health (100) | HS | To cover the fundamental values of health and safety and the current best-practice guidance to adhere to | N/A | Face-to-Face Learning | Theory | Not mentioned |
| Fifth Advanced Course on Health Financing for Universal Coverage for Low and Middle Income Countries | WHO (101) | HS | To analyze and reflect on participants’ own health systems performance, assess the problems it faces, and discuss ideas with professionals from a wide range of countries. | Professional Personnel | Face-to-Face Learning | Theory | Not mentioned |
| **West Bank and Gaza** | | | | | | | |
| Psychosocial training | International Medical Corps (102) | MH | To training volunteers to support children traumatised by the war | Professional Personnel | Face-to-Face Learning | Interactive | Not mentioned |
| An Introduction to Global Health and Its Relevance to Palestine | The Institute of Community and Public Health (ICPH)-Birzeit University in collaboration with PCRF (103) | GH | To explore the links between health and ‘globalization’ (intended as global economic governance) so that implications for policy-making and roles of people’s participation may be discussed, with due attention to the Palestinian geo-political context. | Professional Personnel | Face-to-Face Learning | Theory | Not mentioned |
| Training on implementation of Mother and Child Health e-Registry | The Palestinian National Institute of Public Health (104) | HS | N/A | Professional Personnel | Face-to-Face Learning | Theory | Not mentioned |
| Training of Trainers (TOT) workshop by the Mother and Child Health e-Registry team | The Palestinian National Institute of Public Health (105) | HS | To train core teams at district level on the MCH e-Registry; the teams will facilitate further trainings at the districts level as needed. | Professional Personnel | Face-to-Face Learning | Theory | Not mentioned |
| Newborn care Guidelines Dissemination Workshop | The Palestinian National Institute of Public Health (106) | CH | N/A | Professional Personnel | Face-to-Face Learning | Theory | Not mentioned |
| Multidisciplinary training for primary care – the MA in child health | Royal College of Paediatrics and Child Health Global (107) | CH | To train doctors and nurses to receive a Diploma in Palestinian Child Health (DPCH) | Professional Personnel | Face-to-Face Learning | Interactive | Not mentioned |
| Community Mental Health Training | WHO (108) | MH | To improve and expand the current mental health services, according to a community mental health approach, at the primary, secondary and tertiary levels of health care. | Mixed | Face-to-Face Learning | Practice | Not mentioned |
| WHO's mental health gap action program | WHO (109) | MH | To train the staff in the full range of mental health and psychosocial support services, including school mental health counselling, primary care detection of common mental health problems and rehabilitation skills for mental health staff. | Professional Personnel | Face-to-Face Learning | Theory | European Union |
| **Yemen** | | | | | | | |
| Support for effective stewardship in the Yemeni health sector | EPOS Health Management (110) | HS | To support capacity development of the ministry of public health and population at the individual, organisation, and system levels to lead in the performance of core functions. | Professional Personnel | Blended Learning | Theory | European Union |
| Community-based support program (CBSP) Yemen | Blumont / IRD (111) | Refugee Support | To use community mobilization and cash assistance to serve vulnerable refugees and asylum-seekers in Sana'a. To build community mobilization, by facilitating capacity-building trainings for refugee community leaders to ensure the community's access to comprehensive and effective services. | General Public | Face-to-Face Learning | Theory | UNHCR |
| Health reform support project (hrsp) | Government of Yemen (112) | SRH | 1) To improve access to and quality of priority national family health and reproductive health programs;  2) To develop the capacity of the ministry of public health and population to manage, plan, and deliver basic health services and priority public health programs at the central level and in eight selected districts in the three-targeted governorates. | Professional Personnel | Face-to-Face Learning | Theory | World bank |
| Social protection for community resilience | Social fund for development / UNDP (113) | HS | To improve access to healthcare services and infrastructure through community-based projects and provision of equipment  To strength the ability of local authorities to deliver basic services through better planning, coordination and monitoring | Professional Personnel | Face-to-Face Learning | Theory | European Union |
| Yemen Emergency Crisis Response Project | Social fund for development / public works project / UNDP (114) | SRH | To develop skills of participants to deliver key health and education services | General Public | Face-to-Face Learning | Theory | USAID |
| Yemen emergency health nutrition project | WHO / UNICEF (115) | NCD | To strengthen the delivery of basic health and essential nutrition and water and sanitation services. | Professional Personnel | Not mentioned | Not mentioned | International development association |
| Yemen Field Epidemiology Training Program | Training Programs in Epidemiology and Public Health Interventions Network (116) | EPI | To reduce the burden of major public health problems by training a cadre of public health workers who can provide essential epidemiologic services to the country. | Professional Personnel | Face-to-Face Learning | Practice | Not mentioned |
| Trainings | International Medical Corps (117) | GH | To equip participants with the knowledge and skills required to deliver lifesaving care where it is most needed. | Mixed | Face-to-Face Learning | Theory | Not mentioned |
| GHD/EMPHNET Forms a Team of Trainers on WASH Within Emergency Rapid Response | EMPHNET (118) | CD | To enhance the capacity of participants, enabling them to better assess develop, plan, implement, and monitor WASH-related responses to public health emergencies in Yemen with considerations to humanitarian principles. | Professional Personnel | Face-to-Face Learning | Theory | GHD/EMPHNET |
| Yemen Field Epidemiology Training Program (Y-FETP) Launches Fourth Cohort | The Eastern Mediterranean Public Health Network (119) | EPI | To train participants in field epidemiology training | Professional Personnel | Face-to-Face Learning | Theory | Not mentioned |
| Yemen Training health staff in war surgery | ICRC (120) | DE | To enhance participants capacity to provide life-saving surgical assistance for weapon - wounded casualties and to respond to specific health needs arising in conflicts or other violence. | Professional Personnel | Face-to-Face Learning | Theory | Not mentioned |
| Training on Infection Prevention and Control | WHO - Health Cluster (121) | CD | N/A | Professional Personnel | Face-to-Face Learning | Theory | Not mentioned |
| Training in reproductive health and health education | GIZ GmbH - Deutsche Gesellschaft für Internationale Zusammenarbeit (122) | SRH | To to provide low-threshold access to information and modern contraceptives | Community Workers | Face-to-Face Learning | Theory | Not mentioned |
| UNICEF trained on protection from sexual exploitation and abuse | UNICEF (123) | SRH | UNICEF Staff and partners trained on protection from sexual exploitation and abuse | Professional Personnel | Face-to-Face Learning | Theory | Not mentioned |
| Pre-Service Community Midwifery Training | Yemen Basic Health Services Project (124) | SRH | To increase the number of trained providers by supporting the pre-service training of community midwives | Professional Personnel | Face-to-Face Learning | Theory | USAID |
| Community-based management of acute malnutrition (CMAM) Training | UNICEF (125) | NCD | To provide nutrition services as part of the primary healthcare package and the integration of nutrition services in outreach programs | Professional Personnel | Face-to-Face Learning | Theory | Not mentioned |
| Training in post-abortion care | ICRC (126) | SRH | To train on the application of MVA, counselling on post-abortion family planning, infection identification and prevention and key messages that placed the provision of post-abortion care within the context of preventing maternal deaths | General Public | Face-to-Face Learning | Theory | Not mentioned |
| Regular Training | International Medical Corps (127) | GH | To train participants on health, nutrition, WASH, food security and protection issues | Professional Personnel | Face-to-Face Learning | Theory | Not mentioned |
| **Multiple countries** | |  |  |  |  |  |  |
| International Training on Disaster Medicine | IFMSA (128) | DE | To provide medical students with basic knowledge and introduction to the speciality of Disaster Medicine and create understanding of the disaster management system To provide a platform for students to discuss their perceptions of disaster medicine’s general and specific needs, especially in humanitarian issues, and use the opportunity to analyse medical ethical dilemmas in disaster settings To provide a platform for participants of IFMSA – CRIMEDIM Training for Disaster Medicine Training (TdmT) held in Novara, Italy to train medical students using peer-to-peer methodology by sharing knowledge and skills learned during the TdmT | Professional Personnel | Face-to-Face Learning | Interactive | Not mentioned |
| Health effects and nutrition on emergencies: an introduction | Save the Children, HLA, Kaya (129) | NCD | To train participants on the health and nutrition impacts of emergencies addressed to humanitarian, field, health, and professional workers. | Mixed | Online Learning | Not mentioned | Not mentioned |
| Health effects and nutrition on emergencies: An advanced guide | Save the Children, HLA, Kaya (130) | NCD | To help health and nutrition professionals better anticipate emergencies in the areas of health and nutrition | Mixed | Online Learning | Not mentioned | Not mentioned |

**Abbreviations**

MH: Mental Health

NCD: Non-communicable Diseases

GH: Global Health

SRH: Sexual and Reproductive Health

CH: Child Health

EPI: Epidemiology

DE: Disaster & Emergency

HS: Health Systems

CD: Communicable Diseases

ODH: Oral & Dental Health

IFMSA: International Federation of Medical Students’ Association

PHC: Primary Health Care

HCPs: Health Care Professionals (practitioners, workers, etc.)

MOH: Ministry of Health

MoPH: Ministry of Public Health

ICRC: International Committee of the Red Cross

UNICEF: United Nations Children’s Fund

UNDP: United Nations Development Program

WHO: World Health Organization

CDC: Centers for Disease Control and Prevention

HLA: Humanitarian Leadership Academy

GHD:

EMPHNET: Eastern Mediterranean Public Health Network

1. IFMSA. THEIR FIGHT STORY – ALGERIA (LE SOUK) [Available from: <http://activities.ifmsa.org/their-fight-story-algeria-le-souk/>.

2. IFMSA. WHAT DO YOU KNOW ABOUT HUMAN RIGHTS ? – ALGERIA (LE SOUK) [Available from: <http://activities.ifmsa.org/what-do-you-know-about-human-rights-algeria-le-souk/>.

3. IFMSA. You are important for us … Examine and reassure us [Available from: <http://activities.ifmsa.org/%d8%a3%d9%86%d8%aa-%d8%aa%d9%87%d9%85%d9%8a%d9%86%d8%a7-%d8%a7%d9%81%d8%ad%d8%b5%d9%8a-%d9%88%d8%b7%d9%85%d9%86%d9%8a%d9%86%d8%a7-you-are-important-for-us-examine-and-reassure-us-algeria-le/>.

4. IFMSA. MEDICINE IS NOT CANDY – ALGERIA (LE SOUK) [Available from: <http://activities.ifmsa.org/medicine-is-not-candy-algeria-le-souk/>.

5. IFMSA. MA SOUFFRANCE N’EST PAS DRÔLE #MANICH_MAHBOUL (MY SUFFERING IS NOT FUNNY #IM_NOT_CRAZY) – ALGERIA (LE SOUK) [Available from: <http://activities.ifmsa.org/ma-souffrance-nest-pas-drole-manich_mahboul-my-suffering-is-not-funny-im_not_crazy-algeria-le-souk/>.

6. Reduction UOfDR. UN helps Algeria strengthen disaster risk reduction 2015 [Available from: <https://www.unisdr.org/archive/42375>.

7. Nordisk N. Partnering to innovate diabetes care in Algeria 2016 [Available from: <https://www.novonordisk.com/content/dam/Denmark/HQ/sustainablebusiness/performance-on-tbl/more-about-how-we-work/Creating%20shared%20value/PDF/Partnering-to-innovate-diabetescare-Algeria-BCP-2016-UK.pdf>.

8. Health AAHaMo. Fighting Acute Malnutrition in Djibouti 2011 [Available from: <https://ec.europa.eu/echo/field-blogs/stories/fighting-acute-malnutrition-djibouti_fr>.

9. IFMSA. NO SLUM AREAS (NOSA) – EGYPT (IFMSA-EGYPT) [Available from: <http://activities.ifmsa.org/no-slum-areas-nosa-egypt-ifmsa-egypt/>.

10. IFMSA. Breast Cancer "Pink October" Egypt (IFMSA-Egypt) [Available from: <http://activities.ifmsa.org/breast-cancer-pink-october-egypt-ifmsa-egypt/>.

11. IFMSA. WORLD AIDS CAMPAIGN (WAC) – EGYPT (IFMSA-EGYPT) [Available from: <http://activities.ifmsa.org/world-aids-campaign-wac-egypt-ifmsa-egypt/>.

12. IFMSA. Child Abuse Prevention - Egypt (IFMSA - Egypt) [Available from: <http://activities.ifmsa.org/child-abuse-prevention-egypt-ifmsa-egypt/>.

13. IFMSA. Anti-Microbial Resistance - Egypt (IFMSA - Egypt) [Available from: <http://activities.ifmsa.org/anti-fgm-egypt-ifmsa-egypt/>.

14. IFMSA. Adolescents' Health - Egypt (IFMSA - EGYPT) [Available from: <http://activities.ifmsa.org/adolescents-health-egypt-ifmsa-egypt/>.

15. IFMSA. MSSA 11TH ANNUAL CONFERENCE – DISASTER MEDICINE IN EGYPT – EGYPT (IFMSA-EGYPT) [Available from: <http://activities.ifmsa.org/mssa-11th-annual-conference-disaster-medicine-in-egypt-egypt-ifmsa-egypt/>.

16. IFMSA. Home-Mates - Egypt (IFMSA - Egypt) [Available from: <http://activities.ifmsa.org/home-mates-egypt-ifmsa-egypt/>.

17. IFMSA. Anti-Sexual Harassment - Egypt (IFMSA - Egypt) [Available from: <http://activities.ifmsa.org/anti-sexual-harassment-egypt-ifmsa-egypt/>.

18. IFMSA. ANTI-FGM Egypt (IFMSA-EGYPT) [Available from: <http://activities.ifmsa.org/anti-fgm-egypt-ifmsa-egypt/>.

19. IFMSA. MR. & MS. BREASTESTIS – EGYPT (IFMSA-EGYPT) [Available from: <http://activities.ifmsa.org/mr-ms-breastestis-egypt-ifmsa-egypt/>.

20. IFMSA. MENTAL HEALTH – EGYPT (IFMSA-EGYPT) [Available from: <http://activities.ifmsa.org/mental-health-egypt-ifmsa-egypt/>.

21. EMPHNET T. Egypt Field Epidemiology Training Program (E-FETP).

22. USAID. Strengthening Egypt's family Planning Program 2014 [Available from: <https://www.usaid.gov/egypt/global-health>.

23. Network TEMPH. Egypt’s Ministry of Health and Population Completes Fourth Subnational Emergency Rapid Response Teams Training Workshop 2018 [Available from: <http://emphnet.net/?news=egypts-ministry-of-health-and-population-completes-fourth-subnational-emergency-rapid-response-teams-training-workshop>.

24. Network TEMPH. GHD/EMPHNET Supports Capacity Building for Rapid Response Teams in Egypt. 2018.

25. Network TEMPH. Egypt Ministry of Health and Population Conducts Workshop Two of Public Health Empowerment Program – Basic Field Epidemiology (PHEP-BFE) 2018 [Available from: <http://emphnet.net/?news=egypt-ministry-of-health-and-population-conducts-public-health-empowerment-program-basic-field-epidemiology-phep-bfe-workshop-2>.

26. States ICotRCLoA. Egypt: ICRC and LAS hold training on sexual violence in armed conflicts 2016 [Available from: <https://www.icrc.org/en/document/sexual-violence-ihl-training-Egypt>.

27. Cairo AUi. International Refugee Law [Available from: <https://reliefweb.int/training/3365943/international-refugee-law-january-26-30-2020>.

28. Center TNPHM. Training [Available from: <http://www.whogis.com/workforcealliance/members_partners/member_list/tbzmed/en/>.

29. Migration IOf. IOM Organizes Migration and Development Training for Iranian Officials 2012 [Available from: <https://www.iom.int/news/iom-organizes-migration-and-development-training-iranian-officials>.

30. IFMSA. SEXUALLY TRANSMITTED DISEASES INCLUDING AIDS CAMPAIGN – IRAQ – KURDISTAN (IFMSA-KURDISTAN) [Available from: <http://activities.ifmsa.org/sexually-transmitted-diseases-including-aids-campaign-iraq-kurdistan-ifmsa-kurdistan/>.

31. IFMSA. THE ANTI-SMOKING PROJECT – JORDAN (IFMSA-JO) [Available from: <http://activities.ifmsa.org/the-anti-smoking-project-jordan-ifmsa-jo/>.

32. Network TPiEaPHI. Iraq Field Epidemiology Training Program 2010 [Available from: <https://www.tephinet.org/training-programs/iraq-field-epidemiology-training-program>.

33. Health Mo. Gender-based violence for medical and health staff working in the City of Medicine department 2019 [Available from: <http://www.phd.iq/News_Details.php?ID=3080>.

34. Iraq MoHi. Preparatory workshop on the project of strengthening capacities in the fiels of applied epidemiology 2019 [Available from: <http://www.phd.iq/News_Details.php?ID=3035>.

35. UNFPA GHC, & Avenir Analytics. Strengthening Global Capacity for Emergency Health 2019 [Available from: <https://reliefweb.int/sites/reliefweb.int/files/resources/iraq_hq_bulletin_januray_2019.pdf>.

36. Center CD. From Baghdad to Rochester, International Fellowships Change Lives 2015 [Available from: <https://www.crdfglobal.org/spotlights/baghdad-rochester-international-fellowships-change-lives>.

37. Wisconsin MCo. Global Health Efforts in Asia.

38. Corps IM. Training First Responders in Mosul 2016 [Available from: <https://www.internationalmedicalcorps.org.uk/golden-hour>.

39. Corps IM. Community Health Training 2018 [Available from: <https://internationalmedicalcorps.org/country/iraq/>.

40. Corps IM. Mental Health and Psychosocial Support Training. 2018.

41. IFMSA. It's on us - Jordan (IFMSA - JO) [Available from: <http://activities.ifmsa.org/its-on-us-jordan-ifmsa-jo/>.

42. IFMSA. Here I Am: National Mental Health Project - Jordan (IFMSA - JO) [Available from: <http://activities.ifmsa.org/here-i-am-national-mental-health-project-jordan-ifmsa-jo/>.

43. Network TPiEaPHI. Jordan Field Epidemiology Training Program 2007 [Available from: <https://www.tephinet.org/training-programs/jordan-field-epidemiology-training-program>.

44. Corps IM. mental health and psychosocial protection [Available from: <https://internationalmedicalcorps.org/country/jordan/>.

45. Corps IM. Psychosocial Support, Youth Empowerment and Protection [Available from: <https://internationalmedicalcorps.org/country/jordan/>.

46. EMPHNET, CDC., WHO., UNICEF., IRC. EMPHNET Conducts Workshop to Build a Pool of Potential Consultants for Strengthening Routine Immunization at the Regional Level 2016 [Available from: <http://emphnet.net/?news=emphnet-conducts-workshop-to-build-a-pool-of-potential-consultants-for-strengthening-routine-immunization-at-the-regional-level>.

47. EMPHNET. EMPHNET Holds Pre-Conference Workshop on “Contemporary Issues on Public Health in Emergency” 2015 [Available from: <http://emphnet.net/?news=emphnet-holds-pre-conference-workshop-on-contemporary-issues-on-public-health-in-emergency>.

48. Center NIoH-FI. Fogarty project provided emergency trauma training to Iraqis 2010 [Available from: <https://www.fic.nih.gov/News/GlobalHealthMatters/Pages/0210_trauma-iraq.aspx>.

49. Security UDoSa. Safe and Secure Approaches in Field Environments (SSAFE) Training Course 2018 [Available from: <https://reliefweb.int/training/2928854/safe-and-secure-approaches-field-environments-ssafe-training-course>.

50. IFMSA. ORPHANAGE HEALTH DAY [Available from: <http://activities.ifmsa.org/orphanage-health-day/>.

51. Campaign FS. Food Safety Campaign - Lebanon (LEMSIC) [Available from: <http://activities.ifmsa.org/food-safety-campaign-lebanon-lemsic/>.

52. IFMSA. International Training on Disaster Medicine - Lebanon (LEMSIC) [Available from: <http://activities.ifmsa.org/international-training-on-disaster-medicine-lebanon-lemsic/>.

53. ABAAD. The Concepts Of Srhr, Child Marriage And Working With Adolescents 2016 [Available from: <https://www.daleel-madani.org/civil-society-directory/abaad-dimensions-resource-center-gender-equality/events/training-concepts>.

54. ABAAD. Comprehensive Training On Gender Based Violence Case Management In Emergencies (Gbv Cmie) 2016 [Available from: <https://www.daleel-madani.org/civil-society-directory/abaad-dimensions-resource-center-gender-equality/events/call-participation>.

55. HIVOS A. Training on how to organize support groups for women survivors of gbv using a survivor-centered approach 2015 [Available from: <https://www.daleel-madani.org/civil-society-directory/abaad-dimensions-resource-center-gender-equality/events/abaad-hivos-call-0>.

56. ABAAD. How To Engage Front-Line Workers In Combating Child Marriage 2015 [Available from: <https://www.daleel-madani.org/civil-society-directory/abaad-dimensions-resource-center-gender-equality/events/training-how-0>.

57. Health LMAfS. The LGBT Mental Health Training. 2018.

58. SIDC S. ”Sila” Young People and Drugs: A new Participatory Approach for Lebanon 2016 [Available from: <https://www.socialjusticelebanon.eu/projects/young-people-and-drugs-a-new-participatory-approach-for-lebanon/>.

59. Lebanon NMHPi. Building the capacity of emergency room staf on managing psychitric emergencies 2017 [Available from: <https://www.moph.gov.lb/userfiles/files/Programs%26Projects/MentalHealthProgram/NMHPNewsletterIssue(10).pdf>.

60. Lebanon MoPHi. Integration Of Non-Communicable Disease Services Within Primary Health Care. 2015.

61. Farhood L, Fares S, Hamady C, Maalouf F. Promoting psychosocial and mental health care through community-based educational workshops for teachers and parents. Mental Health and Family Medicine 2018;13:772-81.

62. Lebanon MoPHi. A training course on the surveillance and response system for communicable diseases in health centers and clinics. 2014.

63. Lebanon MoH. Reproductive Health Services 2001 [Available from: <https://www.moph.gov.lb/en/Pages/6/758/reproductive-health-services>.

64. Lebanon Moph. Integration of Non-Communicable Diseases Initiative within Primary Health Care Services 2014 [Available from: <https://www.moph.gov.lb/en/Pages/17/769/integration-of-non-communicable-disease-program-in-primary-health-care>.

65. Lebanon MoPHi. Training Workshop for Nurses with Order of Nurses in Lebanon. 2014.

66. Lebanon MoPH. Jabak Launched the Proficiency Testing Training Project under the AMR Program. 2019.

67. Cross ICotR. Regional training to strengthen response to humanitarian needs held in Beirut 2019 [Available from: <https://www.icrc.org/en/document/regional-training-strengthen-response-humanitarian-needs-held-beirut>.

68. Corps IM. Addressing Regional Mental Health Needs and Gaps in the Context of the Syria Crisis Lebanon2014 [Available from: <https://internationalmedicalcorps.org/wp-content/uploads/2017/07/Syria-Crisis-Addressing-Mental-Health.pdf>.

69. Monde Md. Mental health research among Syrian refugees in Lebanon: challenges and solutions 2018 [Available from: <https://odihpn.org/magazine/mental-health-research-among-syrian-refugees-in-lebanon-challenges-and-solutions/>.

70. AUB. Nutrition in Emergencies Regional Training 2019 [Available from: <http://www.nietraining.net/>.

71. Corps IM. Community Health Training 2017 [Available from: <https://internationalmedicalcorps.org/country/lebanon/>.

72. EMPHNET. EMPHNET Participates in a Workshop on Responding to Refugees’ Health Needs at American University of Beirut Lebanon 2018 [Available from: <http://emphnet.net/?news=21914>.

73. GHI. Mobile University for Health Women's Health Certificate 2019 [Available from: <https://ghi.aub.edu.lb/muh/>.

74. (IHME) GHIHMaE. Global Burden of Disease Training Lebanon2018 [Available from: <https://ghi.aub.edu.lb/event/global-burden-of-disease-training/>.

75. Foundation GHIDN. Hostile Environment Surgical Training Course 2018 [Available from: <https://ghi.aub.edu.lb/event/hostile-environment-surgical-training-course/>.

76. Centre GHIGIoGGH. Global Health Diplomacy Workshop. 2017.

77. Libya MoH. Training of Health Statisticians in the Municipalities of Gammenis and Saloug on the District Health Information System (DHIS2) [Available from: <http://seha.ly/en/2018/12/05/training-course-on-the-use-of-the-dhis2-in-the-municipality-of-shehat/> <http://seha.ly/en/2018/12/04/training-of-health-statisticians-in-the-municipalities-of-gammenis-and-saloug-on-the-district-health-information-system/>.

78. Libya MoHi. Training of Trainers on District Health Information System (DHIS-2) Benghazi – Libya 2018 [Available from: <http://seha.ly/en/2018/07/31/training-of-trainers-on-district-health-information-system-dhis-2-benghazi-libya/>.

79. Corps IM. WORKING TOGETHER TO END MORBIDITY IN CHILDBIRTH 2017 [Available from: <https://www.internationalmedicalcorps.org.uk/working-together-end-morbidity-childbirth>.

80. EMPHNET. GHD/EMPHNET and Libya National Communicable Disease Center Conduct RRT Training of Trainers Workshop 2019 [Available from: <http://emphnet.net/?news=ghdemphnet-and-libya-national-communicable-disease-center-conduct-rrt-training-of-trainers-workshop>.

81. Corps IM. Training in Reproductive Health and Mental Health and Psychosocial Support [Available from: <https://internationalmedicalcorps.org/country/libya/>.

82. Organization WH. Capacity Building to diagnosis and case management for leishmaniasis using WHO and National guidelines 2019 [Available from: <https://www.who.int/health-cluster/countries/libya/Lybia-Health-Sector-Bulletin-July-2019.pdf>.

83. Organization WH. Action for Mental Health Assistance 2019 [Available from: <https://www.who.int/health-cluster/countries/libya/Lybia-Health-Sector-Bulletin-July-2019.pdf>.

84. Libya MoHi. Training of Trainers on District Health Information System (DHIS-2) 28 July - 2nd of August 2018, Tripoli and Benghazi - Libya 2018 [Available from: <https://reliefweb.int/report/libya/training-trainers-district-health-information-system-dhis-2-28-july-2nd-august-2018>.

85. IFMSA. FIGHTING ADDICTION WITH KNOWLEDGE – MOROCCO (IFMSA-MOROCCO) [Available from: <http://activities.ifmsa.org/fighting-addiction-with-knowledge-morocco-ifmsa-morocco/>.

86. Network TPiEaPHI. Morocco Field Epidemiology Training Program 2010 [Available from: <https://www.tephinet.org/training-programs/morocco-field-epidemiology-training-program>.

87. Chevron. Chevron’s Health Partnership in Morocco Makes Progress | Chevron 2015 [Available from: <https://moroccoonthemove.com/2015/07/30/chevrons-health-partnership-morocco-makes-progress-chevron/>.

88. Syria MoHi. Training course on international health regulations 2015 [Available from: <http://www.moh.gov.sy/Default.aspx?tabid=512&language=ar-YE>.

89. Cross ICotR. ICRC trains 57 national society volunteers in water and sanitation 2013 [Available from: <https://www.icrc.org/en/doc/resources/documents/statement/2013/04-24-syria-sarc-water-training.htm>.

90. Corps IM. Addressing Regional Mental Health Needs and Gaps in the Context of the Syria Crisis - Syria Syria 2014 [Available from: <https://internationalmedicalcorps.org/wp-content/uploads/2017/07/Syria-Crisis-Addressing-Mental-Health.pdf>.

91. CDCP. Help and Hope for Syrian Refugees: The Many Ways We Take Action in a Crisis 2012 [Available from: <https://www.cdc.gov/globalhealth/healthprotection/fieldupdates/summer-2016/syrian-crisis-timeline.html>.

92. Corps IM. Mental Health and Psychosocial Support Training [Available from: <https://internationalmedicalcorps.org/country/syria/>.

93. IFMSA. Antimicrobial Resistance Program - Tunisia (Associa-Med) [Available from: <http://activities.ifmsa.org/antimicrobial-resistance-program-tunisia-associa-med/>.

94. IFMSA. SCORA GENERATION – TUNISIA (ASSOCIA-MED) [Available from: <http://activities.ifmsa.org/scora-generation-tunisia-associa-med/>.

95. IFMSA. MENTAL HEALTH PROJECTS – TUNISIA (ASSOCIA-MED) [Available from: <http://activities.ifmsa.org/mental-health-projects-tunisia-associa-med/>.

96. Tunisia MoHi. International course on maternal and neonatal health focused on reducing maternal mortality 2018 [Available from: <http://www.santetunisie.rns.tn/fr/toutes-les-actualites/819-onfp-organise-du-19-novembre-au-1er-d%C3%A9cembre-2018,-un-cours-international-au-profit-des-cadres-m%C3%A9dicaux-et-param%C3%A9dicaux-du-mali-sur-les-%C2%AB-la-sant%C3%A9-maternelle-et-n%C3%A9onatale-ax%C3%A9e-sur-la-r%C3%A9duction-de-la-mortalit%C3%A9-maternelle%C2%BB>.

97. Tunisia MoHi. International training session in reproductive health program management 2018 [Available from: <http://www.santetunisie.rns.tn/fr/toutes-les-actualites/649-session-de-formation-internationale-en-gestion-des-programmes-de-sant%C3%A9-de-la-reproduction>.

98. Tunisie OMdlS. Introduction to Global Health 2019 [Available from: <https://www.facebook.com/events/american-corner-tunis/introduction-to-global-health/1531953073652702/>.

99. Agency JIC. South-South Cooperation: Third Country Training Program 2008 [Available from: <https://www.jica.go.jp/tunisia/english/activities/activity04.html>.

100. Health NEBiOSa. NEBOSH Training | NEBOSH Award In Health And Safety At Work Training in Tunis [Available from: <https://www.theknowledgeacademy.com/tn/courses/nebosh-courses/nebosh-award-in-health-and-safety-at-work/tunis/>.

101. Organization WH. Fifth Advanced Course on Health Financing for Universal Coverage for Low and Middle Income Countries 2018 [Available from: <https://www.who.int/health_financing/training/hfcourse2018/en/>.

102. Corps IM. Mental Health in Gaza 2014.

103. (PCRF) TIoCaPHI-BUicwPCsRF. Global Health training course announcement 2017 [Available from: <https://www.birzeit.edu/en/announcements/global-health-training-course-announcement>.

104. Health TPNIoP. Training on implementation of Mother and Child Health e-Registry [Available from: <https://pniph.org/en/capacity_building>.

105. Health TPNIoP. Training of Trainers (TOT) workshop by the Mother and Child Health e-Registry team [Available from: <https://pniph.org/en/capacity_building>.

106. Health TPNIoP. Newborn care Guidelines Dissemination Workshop [Available from: <https://pniph.org/en/capacity_building>.

107. RCPCH. RCPCH Global: Making a real difference to global child health 2015 [Available from: <https://www.rcpch.ac.uk/sites/default/files/Global_impact_09.04.15.pdf>.

108. WHO. Community Mental Health Development in the occupied Palestinian territory a work in progress with WHO 2001 [Available from: <https://www.mhinnovation.net/sites/default/files/downloads/innovation/reports/Report-Community-Mental-Health-Development-in-Palestine.pdf>.

109. WHO. Health conditions in the occupied Palestinian territory, including east Jerusalem, and in the occupied Syrian Golan 2019 [Available from: <https://apps.who.int/gb/ebwha/pdf_files/WHA72/A72_33-en.pdf>.

110. Yemen: Delivering Health Care Systems Support in a Conflict Zone 2017 [Available from: <https://epos.de/news/current-news/yemen-delivering-health-care-systems-support-conflict-zone>.

111. IRD B. Community-Based Support Program (CBSP) Yemen 2010 [Available from: <https://blumont.org/program/cbsp-yemen/>.

112. Yemen Go. Health Reform Support Project Yemen2011 [Available from: <http://documents.worldbank.org/curated/en/879631475071317358/pdf/000020051-20140624192104.pdf>.

113. UNDP SFfD. Social Protection for Community Resilience 2017 [Available from: <http://www.ye.undp.org/content/yemen/en/home/projects/social-protection-for-community-resilience.html>.

114. UNDP. Yemen Emergency Crisis Response Project (YECRP) - USAID 2019 [Available from: <http://www.ye.undp.org/content/yemen/en/home/projects/yemen-emergency-crisis--response----usaid.html>.

115. UNICEF W. Yemen Emergency Health and Nutrition Project 2019 [Available from: <https://www.worldbank.org/en/news/factsheet/2019/05/14/yemen-emergency-health-and-nutrition-project>.

116. Network TEMPH. Yemen Field Epidemiology Training Program 2017 [Available from: <https://www.tephinet.org/training-programs/yemen-field-epidemiology-training-program>.

117. Corps IM. Capacity Building in Healthcare [Available from: <https://internationalmedicalcorps.org/country/yemen/>.

118. Network TEMPH. GHD/EMPHNET Forms a Team of Trainers on WASH Within Emergency Rapid Response Yemen2018 [Available from: <http://emphnet.net/?news=20055>.

119. Network TEMPH. Yemen Field Epidemiology Training Program (Y-FETP) Launches Fourth Cohort Yemen2018 [Available from: <http://emphnet.net/?news=19975>.

120. Cross ICotR. Yemen: Training health staff in war surgery 2013 [Available from: <https://www.icrc.org/en/doc/resources/documents/news-release/2013/yemen-08-23-war-surgery.htm>.

121. WHO. Yemen: Health Cluster Bulletin, May 2019 2019 [Available from: <https://reliefweb.int/report/yemen/yemen-health-cluster-bulletin-may-2019>.

122. GIZ. Health system strengthening in Yemen 2018 [Available from: giz.de/en/worldwide/17105.html.

123. UNICEF. UNICEF trained on protection from sexual exploitation and abuse 2019 [Available from: <https://www.unicef.org/appeals/yemen.html>.

124. Project YBHS. YEMEN BASIC HEALTH SERVICES (BHS) PROJECT: Mid-Term Evaluation 2009 [Available from: <http://ghpro.dexisonline.com/sites/default/files/resources/legacy/sites/default/files/BHS%20Project%20Mid%20term%20Evaluation_Public.pdf>.

125. UNICEF. Experiences of implementing CMAM in Yemen and number of deaths averted 2018 [Available from: <https://www.ennonline.net/fex/58/cmamyemenaverteddeaths>.

126. ICRC. Family planning and post-abortion care in emergency response – IRC’s experience in Yemen 2014 [Available from: <https://odihpn.org/magazine/family-planning-and-post-abortion-care-in-emergency-response-irc%C2%92s-experience-in-yemen/>.

127. Corps IM. TRAINING TO SAVE LIVES IN YEMEN: IN THE MIDST OF FAMINE. 2018.

128. IFMSA. International Training on Disaster Medicine - IFMSA [Available from: <http://activities.ifmsa.org/international-training-on-disaster-medicine-ifmsa/>.

129. Kaya StC. Health effects and nutrition on emergencies: an introduction [Available from: <https://kayaconnect.org/course/info.php?id=653>.

130. Kaya StC. Health effects and nutrition on emergencies: an advanced guide [Available from: <https://kayaconnect.org/course/info.php?id=654>.
